# Expression of the sRNAs CrcZ and CrcY modulate the strength of carbon catabolite repression under diazotrophic or non-diazotrophic growing conditions in *Azotobacter vinelandii*

**DOI:** 10.1371/journal.pone.0208975

**Published:** 2018-12-13

**Authors:** Marcela Martínez-Valenzuela, Josefina Guzmán, Soledad Moreno, Carlos Leonel Ahumada-Manuel, Guadalupe Espín, Cinthia Núñez

**Affiliations:** 1 Departamento de Microbiología Molecular, Instituto de Biotecnología, Universidad Nacional Autónoma de México (UNAM), Cuernavaca, Morelos, México; 2 Centro de Investigación en Dinámica Celular, Instituto de Investigación en Ciencias Básicas y Aplicadas, Universidad Autónoma del Estado de Morelos (UAEM), Cuernavaca, Morelos, México; Universite Paris-Sud, FRANCE

## Abstract

*Azotobacter vinelandii* is a nitrogen-fixing bacterium of the *Pseudomonadaceae* family that prefers the use of organic acids rather than carbohydrates. Thus, in a mixture of acetate-glucose, glucose is consumed only after acetate is exhausted. In a previous work, we investigated the molecular basis of this carbon catabolite repression (CCR) process under diazotrophic conditions. In the presence of acetate, Crc-Hfq inhibited translation of the *gluP* mRNA, encoding the glucose transporter in *A*. *vinelandii*. Herein, we investigated the regulation in the expression of the small non-coding RNAs (sRNAs) *crcZ* and *crcY*, which are known to antagonize the repressing activity of Hfq-Crc. Our results indicated higher expression levels of the sRNAs *crcZ* and *crcY* under low CCR conditions (i.e. glucose), in relation to the strong one (acetate one). In addition, we also explored the process of CCR in the presence of ammonium. Our results revealed that CCR also occurs under non-diazotrophic conditions as we detected a hierarchy in the utilization of the supplied carbon sources, which was consistent with the higher expression level of the *crcZ*/*Y* sRNAs during glucose catabolism. Analysis of the promoters driving transcription of *crcZ* and *crcY* confirmed that they were RpoN-dependent but we also detected a processed form of CrcZ (CrcZ*) in the RpoN-deficient strain derived from a *cbrB-crcZ* co-transcript. CrcZ* was functional and sufficient to allow the assimilation of acetate.

## Introduction

*Azotobacter vinelandii* is a gamma *Proteobacterium* member of the *Pseudomonadaceae* family. The genus *Azotobacter* is characterized by its ability to develop a differentiation process under adverse growth conditions. This process culminates in the formation of cysts, which are dormant cells resistant to desiccation [1, 2]. *Azotobacter* has a strict aerobic metabolism and is characterized by its capacity to fix atmospheric nitrogen to ammonia under aerobic conditions, with the simultaneous protection of the nitrogenase from oxygen inactivation [2]. The reduction of N_2_ by the nitrogenase is energetically costly. Consequently, the capacity to fix nitrogen is only utilized under conditions of nitrogen limitation.

The energy necessary to sustain nitrogen fixation is derived from the oxidation of carbon sources. *A*. *vinelandii* is a chemo-organotrophic bacterium, that is, it can use many carbohydrates, alcohols and salts of organic acids for growth. However, it is unable to grow using amino acids as the sole carbon source [2]. *A*. *vinelandii* prefers the use of organic acids rather than carbohydrates, hence in a mix of acetate-glucose, acetate prevents the utilization of glucose [3–5]. This preferential use is regulated by the Carbon Catabolite Repression (CCR) that inhibits expression of genes required for degradation/metabolism of the less preferred substrates [6, 7]. In members of the *Pseudomonadaceae* family, the process of CCR is orchestrated by the two-component system CbrA/B and by the post-transcriptional regulatory system Hfq-Crc.

Crc and the RNA chaperone Hfq repress the translation of mRNAs involved in the uptake of non-preferred compounds [8–11]), whereas the CbrA/CbrB two-component system (TCS) activates the transcription of sRNAs of the CrcZ, CrcY or CrcX family in response to the nutritional conditions of the cell. These sRNAs sequester the Crc/Hfq complex counteracting its repressing effect [6, 12–15]. A rich medium elicits a strong CCR response due to a strong activity of Hfq-Crc. Upon relief of CCR, e.g. after exhaustion of the preferred substrate, the levels of the CrcZ-Y-X sRNAs increase leading to sequestration of Hfq/Crc, allowing the utilization of secondary substrates [6, 12, 15]. The nature of the signal detected by the histidine kinase CbrA is not known, but is related to the energetic status of the cell or to the C:N balance. Upon phosphorylation, the response regulator CbrB binds to the regulatory regions of the sRNAs *crcZ* and *crcY*, activating their transcription from RpoN-dependent promoters [6, 12, 13]. The presence of processed forms of CrcZ (named CrcZ*) and CrcY (named CrcY*) in *P*. *putida*, similar in length to the primary ones, was reported [13, 16]. These sRNAs variants come from RNA processing of longer transcripts (*cbrB-crcZ* and PP3539-*mvaB-crcY*) originated from upstream constitutive promoters. CrcZ* was able to antagonize Hfq-Crc, as it relieved the deregulated Hfq-Crc-dependent hyperrepressing phenotype of a Δ*crcZ*Δ*crcY* strain [16]. Thus, CrcZ* is proposed to maintain basal levels of this sRNA protecting the cell from excessive Hfq-Crc-dependent repression.

In a recent work, we reported that under diazotrophic conditions the *A*. *vinelandii* CCR system operates similarly to that present in *Pseudomonas* species. In the diauxic acetate-glucose growth, acetate (the preferred carbon source) was consumed first [5]. Our results indicated that under this condition expression of the glucose GluP transporter was suppressed, but this repression was released once the acetate was exhausted, allowing the uptake of glucose and the growth at the expense of this secondary carbon source. The TCS CbrA/B was essential for the assimilation of glucose, suggesting that expression of the *A*. *vinelandii* sRNAs, *crcZ* and *crcY*, in the diauxic acetate-glucose growth was CbrA/CbrB dependent [5].

In the present work, we investigated the regulation of the sRNAs *crcZ* and *crcY*, during the diauxic acetate-glucose growth under diazotrophic conditions. Our results indicated higher expression levels of the sRNAs *crcZ* and *crcY* under low carbon catabolite repressing conditions (i.e. glucose), in relation to a strong one (acetate) and this response was CbrA/CbrB-dependent. We also explored the process of CCR in the presence of ammonium. Our results revealed that under non-diazotrophic conditions the process of CCR also ocurred as we detected a hierarchy in the utilization of the supplied carbon sources. The analysis of the promoters driving transcription of *crcZ* and *crcY* confirmed they were RpoN-dependent but we also detected a processed form of CrcZ (CrcZ*) in the RpoN-deficient strain, derived from *cbrB-crcZ* co-transcription. We present evidence indicating that CrcZ* was functional and sufficient to alleviate the repressing effect of Hfq-Crc allowing the assimilation of the preferred substrate acetate.

## Materials and methods

### Strains and cultivation conditions

The bacterial strains and plasmids used in the present work are listed in Table 1. The *A*. *vinelandii* wild-type strain AEIV [17] was used in this study.

**Table 1:** Bacterial strains and plasmids used in this study.

Name	Genotype/Relevant characteristics	Reference
AEIV (also named E strain)	Wild type strain	[17]
CFB03	AEIV derivative carries a Sp cassette in the *cbrB* gene (*cbrB*::Sp)	[18]
EQR02	AEIV derivative carries a Sp cassette in the *cbrA* gene (*cbrA*::Sp)	[5]
CN10	ATCC 9046 derivative carries a Gm cassette in the *rpoN* gene (*rpoN*::Gm)	[19]
AErpoN	AEIV derivative carries a Gm cassette in the *rpoN* gene (*rpoN*::Gm)	This work
AE-Zgus	AEIV derivative carries a chromosomal *crcZ*-*gusA* transcriptional fusion. Tc^r^	[5]
AE-Ygus	AEIV derivative carries a chromosomal *crcY*-*gusA* transcriptional fusion. Tc^r^	[5]
AErpoNZgus	*rpoN*::Gm mutation derivative carries a chromosomal *crcZ*-*gusA* transcriptional fusion. Tc^r^	This work
AErpoNYgus	*rpoN*::Gm mutation derivative carries a chromosomal *crcY*-*gusA* transcriptional fusion. Tc^r^	This work
JG513	AEIV derivative carries a chromosomal *cbrB*-*gusA* transcriptional fusion. Tc^r^	This work
AErpoNBgus	*rpoN*::Gm mutation derivative carries a chromosomal *cbrB*-*gusA* transcriptional fusion. Tc^r^	This work
**Plasmids**		
pJET1.2/Blunt vector	Cloning vector; Ap^r^	Thermo Scientific
pJET::P*cbrB*	pJET derivative carrying the *cbrB* regulatory region	This work
pUMATcgusAT	Vector with the *gusA* gene for transcriptional fusions; Ap^r^, Tc^r^	[20]
pUMApcbrB	pUMATcgusAT derivative carrying a *cbrB-gusA* transcriptional fusion	This work
pZPE	Plasmid carrying the regulatory region of *crcZ*	This work

*A*. *vinelandii* was routinely grown in minimal Burk’s medium supplemented with 20g L^-1^ of sucrose (Burk’s-sucrose medium) at 30°C [21]. Burk´s minimum medium supplemented with acetate (30 mM) and glucose (30 mM) (BAG medium) was used for diauxic growth. When indicated, other carbon sources were employed in a final concentration of 30 mM. The composition of the growth medium and culture conditions have been reported elsewhere [22]. *Escherichia coli* DH5 alpha [23] was grown in LB medium at 37°C [24]. When needed, the final antibiotic concentrations (in μg mL^-1^) used for *A*. *vinelandii* and *E*. *coli* were as follows; tetracycline (Tc), 30 and 15; gentamicin (Gm), 1 and 10; spectinomycin (Sp), 100 and 100; ampicillin (Ap), not used and 200.

*A*. *vinelandii* transformation was carried out as described [25]. To ensure double reciprocal recombination and allelic exchange, mutants constructed by reverse genetics were generated by transforming *A*. *vinelandii* cells with linear DNA carrying the desired mutation. At least two independent transformation events were conducted, and transformants were selected using the corresponding antibiotic. Three representative transformants were confirmed by PCR analysis to carry the desired mutation. Due to the polyploid nature of *A*. *vinelandii* [2], the segregation of the generated mutation to all chromosomal copies (and the absence of wild type alleles) was confirmed by PCR. Only one confirmed mutant was chosen for further studies.

### Standard techniques

DNA isolation and cloning were conducted as described [26]. The oligonucleotides used for PCR amplifications were designed based on the DJ *A*. *vinelandii* genome sequence [27]. Sequences of the oligonucleotides used in this study are listed in Table 2. The high fidelity Phusion DNA polymerase (Thermo Scientific) was used for all PCR amplifications and they were confirmed by DNA sequencing. DNA sequencing was done with fluorescent dideoxy terminators using a cycle sequencing method and the 3130xl analyzer of Applied Biosystems.

**Table 2:** Sequences of the primers used in this study.

Primer Name	Nucleotide sequence (5′–3′)	Reference
gyrAfw	CCAGCAAGGGCAAGGTCTA	[22]
gyrArev	TCGTCCAGCGGCAACAGGT	[22]
Zfw1	GAACAACAAAACTGCCACGA	This work
Zrv1	GAGCCAATAGCAAACGGATT	This work
Zfw2	GCCATCCATGTCACACAATC	This work
CrcY qPCR F	CGGACTGGTTGGATCACTTG	This work
CrcY qPCR R	GACCGCCACTCTGAAGAAAG	This work
fusB_Fw_X1	TCTAGAGTGGAACAGAGCATCGACC	This work
fusB_Rv_R1	GAATTCGGATGATGGTTTCGTCTTC	This work
crcZRvPrimerE3	CTGGAGTCGTGTCGTCGTTC	This work
pcrcZFXb	TCTAGAGACCTGGAGGACGATGATTTC	This work
rpoN_Fw	TTACCAGGAAGGCTACGAGA	This work
rpoN_Rv	GAGCCACCTGTATGCCTTGT	This work

### Construction of a RpoN-deficient strain derived from AEIV

Strain CN10, is an *A*. *vinelandii* ATCC 9046 derivative with an insertion of a Gm resistance cassette within the *rpoN* gene (*rpoN*::Gm) [19], and was used to generate an *rpoN*^*-*^ mutant in the background of strain AEIV. This strain was made competent and was transformed with CN10 chromosomal DNA and Gm^r^ transformants were selected. PCR amplification of the *rpoN locus*, using oligonucleotides RpoN_Fw and RpoN_Rw, confirmed the presence of the corresponding construction in the chromosome and its segregation to all chromosomal copies of *A*. *vinelandii*. The resulting strain was named AErpoN.

### Construction of strains carrying a chromosomal *PcbrB*–*gusA* transcriptional fusion

A DNA fragment carrying the promoter region of *cbrB* (*PcbrB*) was PCR amplified using oligonucleotides fusB_Fw_X1 and fusB_Rv_R1 (Table 2). This fragment (335 bp) spanning a region from nt -245 to +30 relative to the first nucleotide of the *cbrB* translation initiation codon, was sub-cloned into the pJET1.2/Blunt vector (Thermo Scientific) generating plasmid pJET::P*cbrB*. *PcbrB* was obtained as an *EcoRI* fragment after digestion of pJET::PcbrB with this endonuclease and was subsequently cloned into vector pUMATcgusAT [20], previously cut with the same enzyme. The generated plasmid was named pUMAPcbrB. The vector pUMATcgusAT is useful for the construction of *gusA* transcriptional fusions that can be directed to the chromosome after a double recombination event within the *melA locus*. A Tc^r^ cassette adjacent to the *gusA* gene served as a selection marker [20]. The wild-type strain AEIV and the AErpoN mutant were transformed with pUMAPcbrB, previously linearized with *NdeI* endonuclease. Double recombinants Tc^r^ were selected, generating strains JG513 (AEIV *PcbrB-gusA*) and AErpoNBgus (*rpoN*^-^, *PcbrB-gusA*), respectively. The presence of the *PcbrB*-*gusA* transcriptional fusions in their chromosomes was confirmed by PCR amplification.

### Construction of RpoN-deficient strains carrying chromosomal *PcrcZ-gusA* or *PcrcY-gusA* transcriptional fusions

Strain AE-Zgus, carrying the transcriptional *PcrcZ-gusA* fusion [5] was transformed with chromosomal DNA from mutant AErpoN (*rpoN*::Gm) and Gm^r^ transformants were selected. The resulting strain was named AErpoNZgus. PCR amplification of the *rpoN locus*, followed by DNA sequencing, confirmed the corresponding construction and its segregation to all the chromosomal copies of *A*. *vinelandii*.

Strain AE-Ygus carrying the transcriptional *PcrcY*-*gusA* fusion [5] was transformed with chromosomal DNA from mutant AErpoN (*rpoN*::Gm) and Gm^r^ transformants were selected. The resulting strain was named AErpoNYgus. PCR amplification of the *rpoN locus*, followed by DNA sequencing, confirmed the corresponding construction and its segregation to all the chromosomal copies of *A*. *vinelandii*.

### Quantitative real-time PCR (qRT-PCR)

Cells of *A*. *vinelandii* were collected by centrifugation, and the total RNA was extracted as described [28]. Genomic DNA contamination was removed with DNase I (Thermo Scientific). Details of cDNA synthesis and qRT-PCR amplification conditions have been reported elsewhere [25]. qRT-PCR assays were performed with a Light Cycler 480 II instrument (Roche), using the Maxima TM SYBR Green/ROX qPCR Master Mix (2X) kit (Thermo Scientific). The relative levels of CrcZ and CrcY were quantified comparing the amounts of each RNA under the tested conditions, using the *gyrA* (Avin15810 or AVIN_RS07245 of the updated annotation) mRNA as an internal control, since expression of this gene is rather constant [22, 25, 29]. The sequences of the primer pairs used for the quantification of the mRNA of *crcZ* (Zfw1 and Zrv1), *crcY* (CrcY qPCR F and crcY qPCR R) and *gyrA* (gyrAfw and gyrArev) are listed in Table 2. These primers were designed using the Primer3 program (http://bioinfo.ut.ee/primer3/) with an optimal length of 20 bases, and a melting temperature of 60°C. Verifying specific single product amplification by melting-curve analyses validated each primer set. Thereafter the efficiency of the PCR was estimated by developing standard curves for each amplicon using dilution series of the cDNA corresponding to the reference sample. cDNAs derived from the reference and experimental samples were amplified using quantities within the linear range of the standard curve. Three biological replicates (independent cultures) were performed with three technical replicates for each one. Similar results were obtained for the transcription of all measured genes in the repetitions. A non-template control reaction was included for each gene. The quantification technique used to analyze the generated data was the 2^-Δ,ΔCT^ method reported previously [30].

### Semi-quantitative RT-PCR of *crcZ*

3μg of the total RNA extracted from the wild type strain AEIV or from its isogenic AErpoN mutant were treated with DNase I (Thermo Scientific), to eliminate genomic DNA. Thereafter, generation of cDNA by reverse transcription was conducted using 200 ng of RNA, the reverse oligonucleotide Zrv1 and the RevertAid H Minus Reverse Transcriptase (Thermo Scientific), as instructed by the manufacturer. Prior to the generation of the cDNA, some RNA samples (500 ng) were treated with Terminator 5’-phosphate-dependent exonuclease enzyme (TEX, Epicentre), which specifically degrades RNAs having a 5’ monophosphate but not the primary transcripts with three phosphates at their 5’ end. Transcripts corresponding to the intergenic *cbrB-crcZ* region or to the *crcZ* gene were detected using the oligonucleotide pairs Zfw2/Zrv1 and Zfw1/Zrv1, respectively, in a 25-cycle PCR program; from 1–20 ng of cDNA were used as a template. A 100 bp *gyrA* amplicon was used to assure the integrity of the cDNA and as internal normalizer during quantification of *crcZ* levels. To this end, 20 ng of cDNA were used as a template and the oligonucleotides pair gyrAfw/gyrArev. The relative levels of *crcZ* transcripts were estimated by densitometry using the ImageJ program [31]. We also performed negative and positive controls using no cDNA or using the AEIV genomic DNA, respectively, as a template. RT-PCR products were resolved by 1% agarose gel electrophoresis and visualized by staining with HydraGreen^TM^ (ACTGene).

### Primer extension assays

The transcriptional start site of *crcZ* was mapped by primer extension analysis. Total RNA was prepared as previously reported [28] from the *A*. *vinelandii* wild-type strain grown in BAG medium, with or without 15 mM NH_4_Cl. The oligonucleotide used as a primer for the extension reaction, named crcZRvPrimerE3 (Table 2), was end-labeled with [γ-^32P^]-ATP and T4 polynucleotide kinase (Roche). Primer extension was performed at 42°C with AMV reverse transcriptase (Roche) as indicated by the supplier. The extended cDNA product was analyzed by electrophoresis on a denaturing 6% urea-polyacrylamide gel, in parallel with a DNA sequence ladder generated with the same primer by using a Thermo Sequenase Cycle Sequencing kit (USB). Plasmid pZPE, carrying the regulatory region of *crcZ*, was used as a DNA template. Plasmid pZPE was constructed by PCR amplification of a 472 bp fragment with oligonucleotides crcZRvPrimerE3 and pcrcZFXb. The product was then ligated to vector pJET1.2/blunt, generating plasmid pZPE.

### Analytical methods

Total Protein quantification was determined by the Lowry method [32]. β-glucuronidase activity was determined as reported elsewhere [33]. One U corresponds to 1 nmol of *O-*nitrophenyl-ß-D-glucuronide hydrolyzed per min per μg of protein

### High performance liquid chromatography

Quantification of glucose and acetate was performed by HPLC using an Aminex HPX-87H column (300 mm × 7.8 mm) (Biorad, Hercules, CA, USA), as reported previously [5]. The eluent was H_2_SO_4_ (7 mM) and was eluted at a flow rate of 0.8 mL min^−1^. Glucose was detected using a refractive index (RI) detector (Waters 2414 detector). The supernatant was also tested for the presence of acetate, using the same chromatographic method, followed by UV absorption at 210 nm using a photodiode array detector (Waters 2996 detector).

### Statistical analysis

Statistical analyses were performed using GraphPad Prism (version 6.0) software (GraphPad Software, La Jolla, CA). Statistical significance was determined using two-tailed, unpaired Student’s t-test, and a p-value ≤0.05 was considered significant.

## Results

### The presence of acetate inhibits the activity of *PcrcZ* and *PcrcY* promoters and the accumulation of the CrcZ and CrcY sRNAs

**Fig 1:**
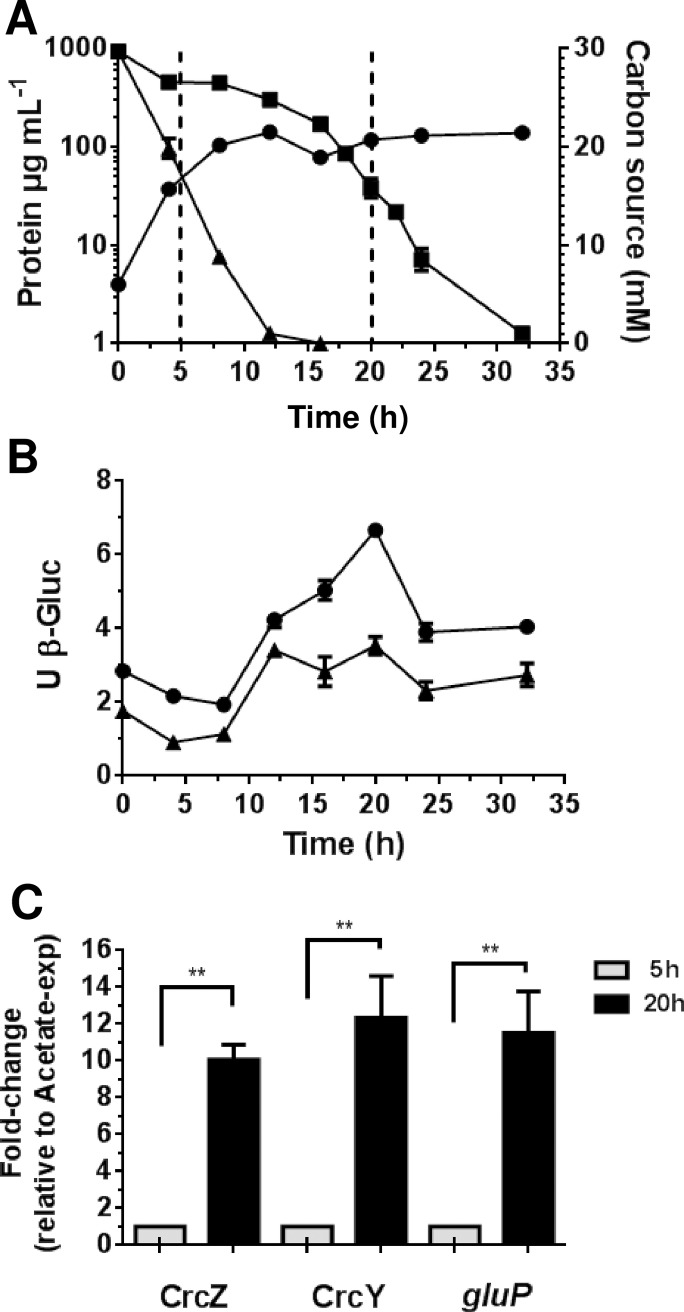
Transcriptional regulation of *crcZ* and *crcY* in diazotrophic conditions. A. Growth kinetic (circles) and acetate (triangles) or glucose (squares) consumption of the *A*. *vinelandii* wild type strain AEIV cultured in Burk’s minimum medium supplemented with 30 mM acetate and 30 mM glucose (BAG medium). B. Activity of the promoters for *crcZ* and *crcY*. Strains AE-Zgus and AE-Ygus, carrying *PcrcZ-gusA* (circles) and *PcrcY-gusA* (triangles) transcriptional fusions, respectively, were cultured in 25 mL of Burk’s medium amended with 30 mM acetate for 12 h; Afterward, the same amount of cell culture (corresponding to 200 μg of protein) was used to inoculate 50 ml of BAG medium. Cells were harvested along the growth curve and the activity of ß-glucuronidase (ß-Gluc) was determined. C. Quantification of CrcZ, CrcY and *gluP* transcripts by qRT-PCR analysis. The total RNA was extracted from cells growing in diauxic BAG medium at the expense of acetate (5h; gray bars) or glucose (20 h; black bars). The bars of standard deviation from three independent experiments are shown. In panel A these bars are not visible since they are smaller than the symbols used. Significant differences were analyzed by *t*-test. Statistical significance is indicated (**p<0.01).

In order to analyze the transcriptional regulation of the sRNAs CrcZ and CrcY, during the diauxic acetate-glucose growth, strains AEZ-gus and AEY-gus were used. These strains are derivatives of the wild type strain AEIV, carrying transcriptional fusions of the *crcZ* and *crcY* promoters (*PcrcZ* and *PcrcY*, respectively) with the *gusA* reporter gene [5]. They were cultured in BAG medium (minimum Burk’s medium with acetate and glucose as carbon sources), and the activity of ß-glucuronidase was measured along the growth curve. As anticipated, during the first 10 h, cell growth occurred at the expense of acetate; thereafter, glucose assimilation started and growth continued until it reached a concentration of approximately 140μg of protein mL^-1^ (Fig 1A).

The activity of both promoters, *PcrcZ* and *PcrcY*, was the lowest during acetate consumption (Fig 1B). In fact, a clear decrease in the activity of the reporter enzyme was observed for both, implying the lack of *de novo* synthesis of ß-glucuronidase and a dilution effect. A sharp induction of *crcZ* and *crcY* expression was observed at the same time as the acetate was being depleted and the glucose utilized, increasing the activity of *PcrcZ* and *PcrcY* by about 3-fold. It should be noted that the activity of *PcrcY* was lower than that of *PcrcZ*. A peak in the activity at around the mid point of glucose consumption was consistently observed for *PcrcZ* but not for *PcrcY* (Fig 1B). Altogether, these data suggested that CrcZ and CrcY sRNAs accumulated under low catabolite repressing conditions.

**Fig 2:**
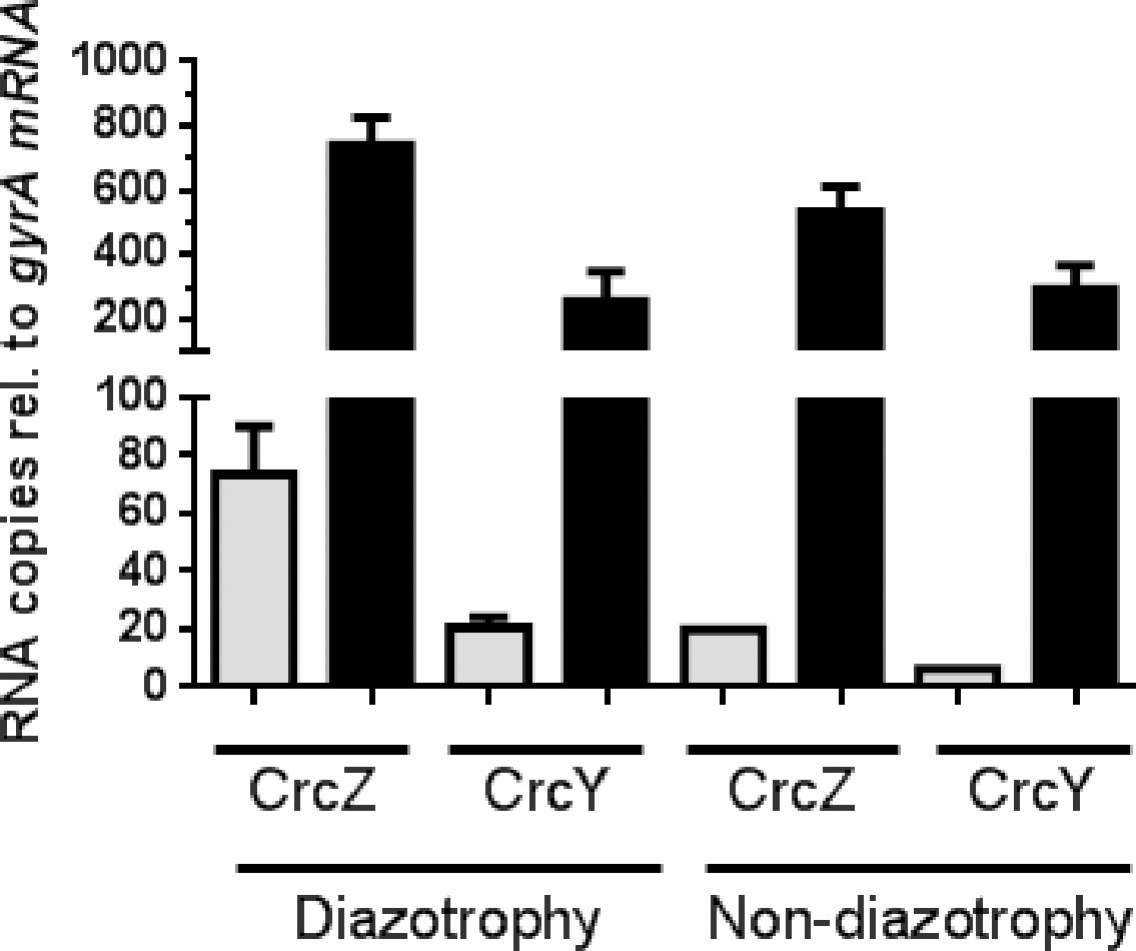
Quantification of the CrcZ and CrcY levels. Relative levels of the CrcZ and CrcY sRNAs, measured by qRT-PCR in cells of the *A*. *vinelandii* AEIV strain growing exponentially at the expense of acetate (gray bars) or glucose (black bars) in BAG medium in the absence (diazotrophy) or presence (non-diazotrophy) of 15 mM NH_4_Cl. The values are expressed as RNA copies relative to those of *gyrA* mRNA (internal standard).

Next, we determined the relative levels of the sRNAs CrcZ and CrcY. To this end, the total RNA, derived from the wild type strain AEIV, was extracted while growing in the diauxic BAG medium at the expense of acetate (5 h) or glucose (20 h); the relative accumulation of the sRNAs was determined by qRT-PCR, using the *gyrA* mRNA as an internal control (see Materials and methods). The levels of both sRNAs increased about 10-fold under glucose-growing conditions with respect to the levels observed in acetate-growing cells (Fig 1C). Furthermore, analysis of the levels of the individual sRNAs showed that CrcZ abundance was 3- to 4-fold higher than that of CrcY either in repressing (acetate) or non-repressing (glucose) conditions (Fig 2), a situation resembling that of *P*. *putida* [16]. This result agrees with the higher activity observed for *PcrcZ* relative to *PcrcY* (Fig 1B). As an internal control, the levels of the *gluP* mRNA, encoding the glucose transporter in *A*. *vinelandii*, were also determined and showed a consistent increase of about 13-fold under glucose assimilation, in agreement with our previous work [5]. Together, these results implied that accumulation of the sRNAs CrcZ and CrcY correlated with the release in the translational repression of the *gluP* mRNA exerted by the Crc-Hfq protein complex, allowing glucose transport and metabolism.

### *A*. *vinelandii* also exhibits diauxie when growing in non-diazotrophic conditions

**Fig 3:**
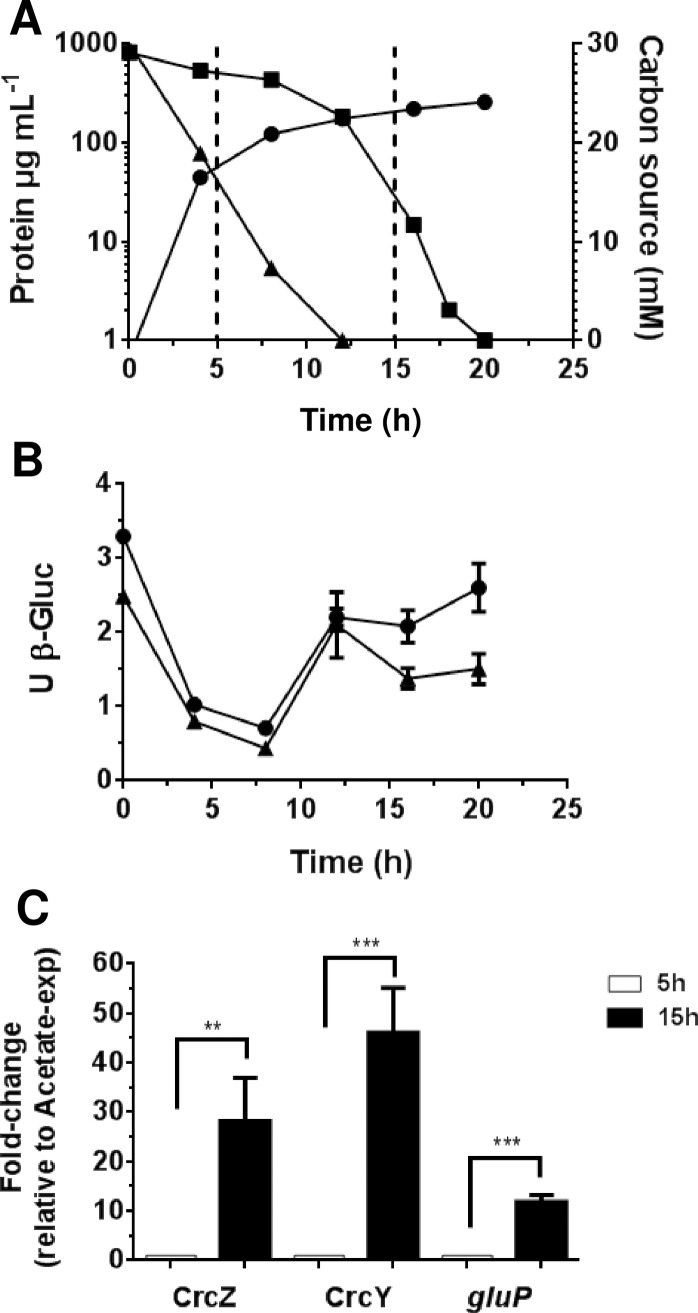
Transcriptional regulation of CrcZ and CrcY in non-diazotrophic conditions. A. Growth kinetic (circles) and acetate (triangles) or glucose (squares) consumption of the *A*. *vinelandii* wild type strain AEIV cultured in Burk’s minimum medium supplemented with 30 mM acetate, 30 mM glucose and 15 mM NH_4_Cl (BAG-N medium). B. Activity of the *crcZ* and *crcY* promoters. Strains AE-Zgus and AE-Ygus, carrying *PcrcZ-gusA* (circles) and *PcrcY-gusA* (triangles) transcriptional fusions, respectively, were cultured in 25 mL of Burk’s medium amended with 30 mM acetate for 12 h; then, 50 ml of BAG medium was inoculated with the same amounts of cells (corresponding to 200 μg of protein). Cells were harvested along the growth curve and the activity of ß-glucuronidase (ß-Gluc) was determined. C. Quantification of CrcZ, CrcY and *gluP* transcripts by qRT-PCR analysis. The total RNA was extracted from cells growing in diauxic BAG-N medium at the expense of acetate (5h; gray bars) or glucose (15 h; black bars). The bars of standard deviation from three independent experiments are shown. In panel A these bars are not visible since they are smaller than the symbols used. Significant differences were analyzed by *t*-test. Statistical significance is indicated (**p<0.01 or ***p<0.0001).

We also explored the regulation of CCR in the presence of ammonium. For this purpose, the wild type strain AEIV strain was cultured in diauxic BAG medium in the presence of 15 mM NH_4_Cl (BAG-N). As shown in Fig 3A, CCR was observed as revealed by the hierarchy in the consumption of the carbon sources. Acetate was consumed during the first 10 h of growth, and once it was depleted, a sharp assimilation of glucose ocurred (Fig 3A). It should be noted that the glucose was totally consumed after 18 h of diauxic growth, at about 5 h earlier than in diazotrophic conditions (Fig 1A). As expected, in the presence of ammonium cell growth was higher than in its absence, reaching a maximum protein concentration of 260μg mL^-1^.

### In non-diazotrophic conditions expression of the sRNAs *crcZ* and *crcY* increase during glucose consumption

The activity of *PcrcZ* and *PcrcY* was evaluated along the diauxic acetate-glucose growth in the presence of ammonium. Their expression pattern was similar to that observed for cells cultured under diazotrophic conditions: during acetate assimilation, the activity of ß-glucuronidase showed a dilution effect implying inactivity of *PcrcZ* and *PcrcY* promoters, while they were activated from 3- to 4-fold during glucose growth (Fig 3B). This result was expected considering the sequential assimilation of the substrates in the culture medium.

qRT-PCR quantification of the relative levels of CrcZ and CrcY at 15 h of diauxic growth, corresponding to the period of glucose consumption, showed that these sRNAs exhibited an increase of 28- and 46-fold, respectively, in relation to the acetate growing condition (5 h) (Fig 3C); such an increase was from 3 to 4-fold higher than in diazotrophy (compared Fig 1C and Fig 3C). Analysis of the relative abundance of the individual sRNAs indicated that the levels of CrcZ and CrcY during glucose growth were rather similar to the levels obtained in the absence of ammonium, and that their different fold change between repressing and non-repressing conditions derived from the lower levels of CrcZ and CrcY under acetate growth in the presence of ammonium (Fig 2). As expected, the relative *gluP* mRNA levels increased 11 times allowing glucose uptake (Fig 3C).

Altogether, these results indicated that in *A*. *vinelandii* CCR operates in a similar way under diazotrophic and non-diazotrophic conditions. In both cases CrcZ and CrcY are required for de-repressing the translational effect of Hfq-Crc on *gluP* mRNA, enabling glucose uptake and metabolism.

### The presence of CrcY, but not that of CrcZ, is totally dependent on CbrA

**Fig 4:**
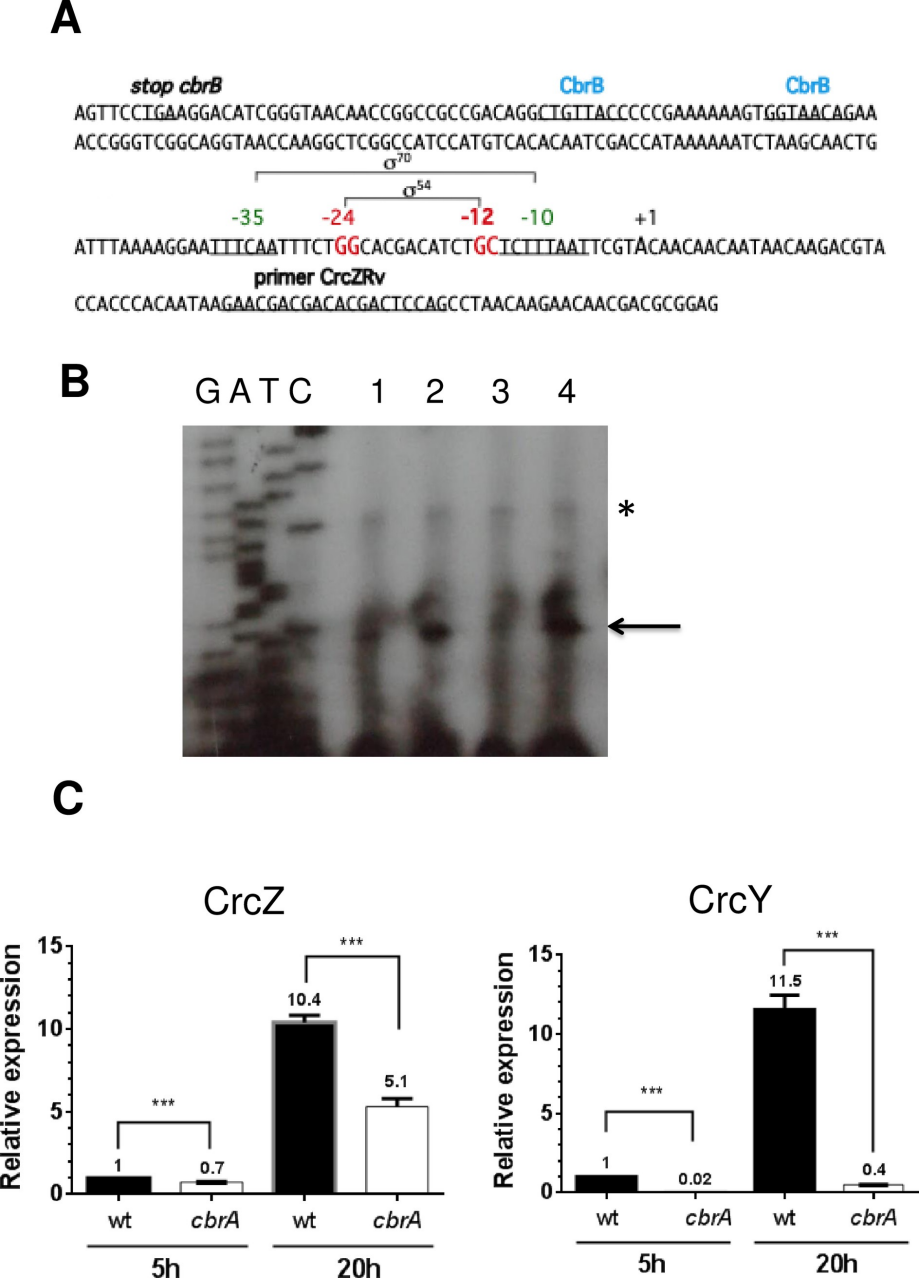
Transcriptional regulation of CrcZ and CrcY sRNAs. A. DNA sequence of the regulatory region of *crcZ*. The transcriptional initiation site is indicated (+1), along with the predicted σ^70^ and σ^54^ promoters. The putative sequences recognized by CbrB are indicated. B. Identification of the transcription initiation site of *crcZ*. The primer extension analysis was conducted with RNA extracted from cells grown in BAG (lanes 1 and 2) or BAG-N (lanes 3 and 4) medium, during growth at the expense of acetate (lanes 1 and 3) or glucose (lanes 2 and 4). A primer complementary to *crcZ* was used and its sequence is indicated in panel A. The cDNA obtained was resolved in a denaturing poly-acrylamide gel, side by side with DNA sequence ladders obtained by chemical sequencing of *crcZ*. The transcriptional initiation site is indicated by an arrow. A second signal, corresponding to a transcript 13 nt longer than the primary one, is also indicated (*). C. Quantification of CrcZ and CrcY transcripts by qRT-PCR analysis in the wild type train AEIV (wt) and in its derivative *cbrA*::Sp mutant EQR02 (*cbrA*), grown in diauxic BAG medium at the expense of acetate (5h) or glucose (20 h). The bars of standard deviation from three independent experiments are shown. Significant differences were analyzed by *t*-test. Statistical significance is indicated (***p<0.001).

A comparative analysis of the *A*. *vinelandii crcZ* and *crcY* regulatory regions with those of *P*. *putida* allowed the identification of conserved residues characteristics of RpoN (σ^54^)-dependent promoters [5]. We also identified conserved sequences recognized by CbrB (Fig 4A). In addition, putative σ^70^ promoters were also detected for *crcZ* and *crcY*, using the BPROM program for bacterial sigma70 promoter recognition (http://linux1.softberry.com/berry.phtml?topic=bprom&group=programs&subgroup=gfindb), which overlap the predicted σ^54^ promoters. This result raised the question about the nature of the functional promoter driving expression of *crcZ* and *crcY* under the diauxic acetate-glucose growth.

Primer extension analyses were conducted to experimentally establish the *crcZ* transcriptional start point in conditions of CCR and non-CCR (i.e. acetate or glucose growth). It was achieved using total the RNA extracted from the wild type strain grown in BAG medium, at the expense of either acetate (5 h) or glucose (20 h). As shown in Fig 4B, the primer extension assay revealed a single start point for *crcZ* thirteen and five bp downstream of the predicted σ^54^ and σ^70^ promoters, when total RNA from acetate or glucose grown cells was used, suggesting that any of these two promoters could be driving the expression of *crcZ*. The primer extension assay was also performed in cells grown in the presence of ammonium. Our results indicated that this condition did not change the transcriptional initiation site of *crcZ*. It should be noted that the amount of the cDNA detected in cells grown in the presence of glucose, with or without ammonium, was higher than that of cells consuming acetate, consistent with our results from the expression analysis of *PcrcZ* and from CrcZ RNA quantification.

Previous studies conducted in *A*. *vinelandii* grown in sucrose as the carbon source, indicated that the activity of *PcrcZ* and *PcrcY* relies on the histidine kinase CbrA, as their expression was reduced in a *cbrA*^*-*^ genetic background, with respect to the wild-type strain [5]. In order to confirm the essential role of CbrA on *crcZ* and *crcY* expression, the amount of these sRNAs was determined in a *cbrA* genetic background (*cbrA*::Sp). The wild type strain AEIV and its *cbrA*^*-*^ derivative were cultured in BAG medium and the total RNA was extracted during acetate or glucose growth. As seen in Fig 4C, CbrA showed a partial effect on the accumulation of CrcZ, as its levels were not suppressed, but were reduced down to 50% in the CbrA-deficient strain in the presence of glucose. In contrast, transcriptional activation of CrcY was totally dependent on CbrA as the levels of this sRNA were abrogated in the *cbrA*^*-*^ mutant. These results indicated that a RpoN promoter activated by the two-component system CbrA/CbrB was driving *crcY* expression while expression of *crcZ* was only partially dependent on this sigma factor.

### RpoN drives transcription from *PcrcZ* and *PcrcY*

In order to explore the dependence of *crcZ* and *crcY* expression on the RpoN sigma factor, we constructed a RpoN-deficient strain, named AErpoN, and evaluated its growth phenotype in the diauxic BAG medium.

**Fig 5:**
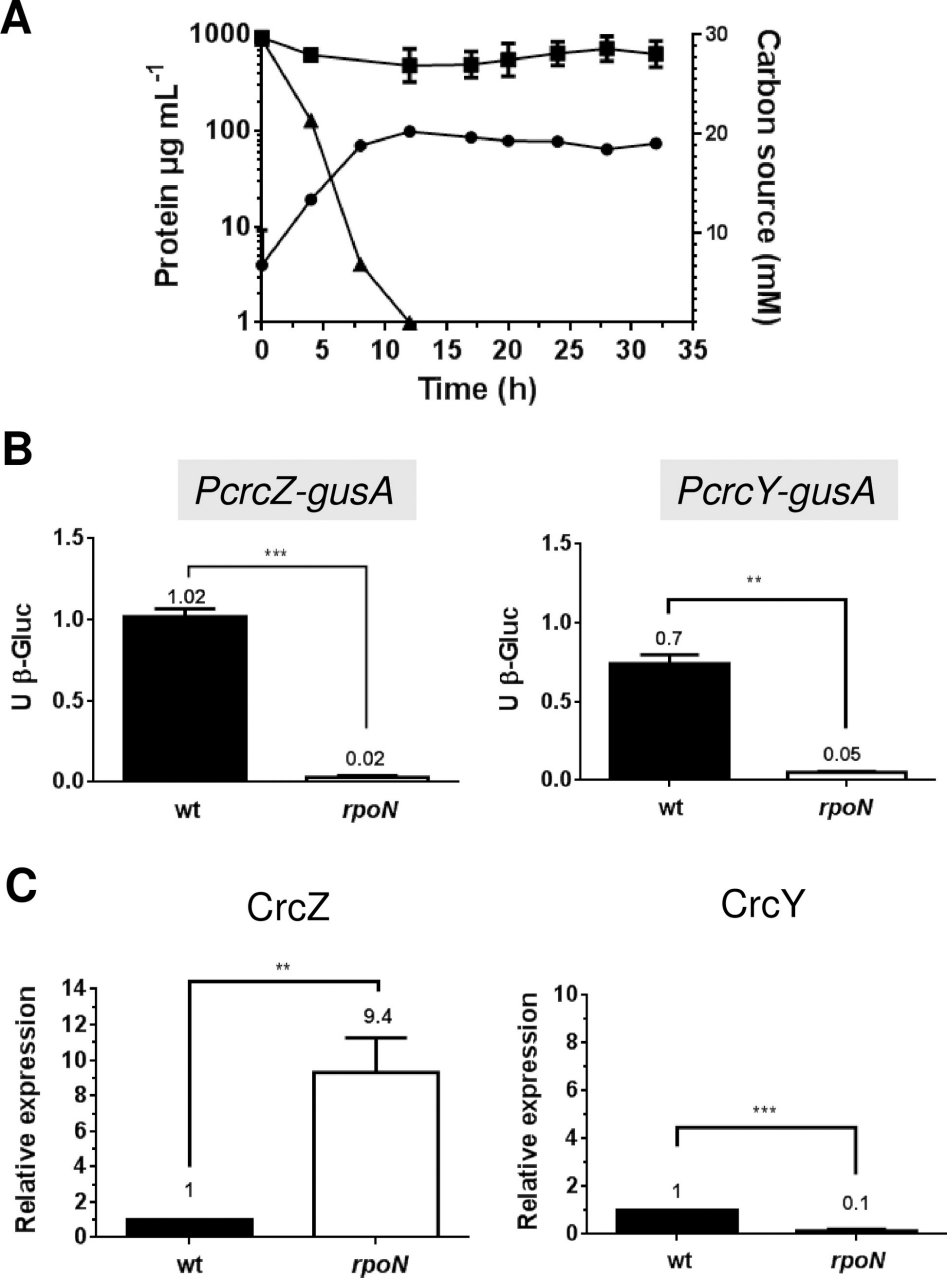
Transcriptional regulation of CrcZ and CrcY by RpoN. A. Growth kinetic (circles) and acetate (triangles) or glucose (squares) consumption by the AErpoN (*rpoN*::Gm) strain cultivated in BAG-N medium. B. The activity of *PcrcZ* and *PcrcY* is RpoN-dependent. Strains AE-Zgus (*PcrcZ-gusA*) (wt) and AE-Ygus (*PcrcY-gusA*) (wt), and their respective *rpoN*::Gm derivatives (*rpoN*) were cultured in 25 mL of Burk’s medium amended with 30 mM acetate for 12 h, afterward 200 μg of protein derived from these pre-inoculums were used to inoculate 50 ml of BAG-N medium. Cells were harvested under acetate growing conditions (5h) and the activity of ß-glucuronidase (ß-Gluc) was determined. C. Quantification of CrcZ and CrcY transcripts by qRT-PCR analysis in the wild type strain AEIV (black bars) or in AErpoN strain (white bars). The total RNA was extracted from cells growing in diauxic BAG-N medium at the expense of acetate (5h). The bars of standard deviation from three independent experiments are shown. Significant differences were analyzed by *t*-test. Statistical significance is indicated (**p<0.01 or ***p<0.0001).

As anticipated, based on the role of this sigma factor in nitrogen metabolism, this mutant was unable to grow diazotrophically. Therefore, the ability of the AErpoN strain to overcome CCR and grow at the expense of glucose was studied under diauxic acetate-glucose conditions in the presence of ammonium. As shown in Fig 5A, this mutant showed wild-type growth kinetic using acetate as a carbon source, during the first 12 h. However, once acetate was depleted, growth stopped as this mutant was unable to utilize glucose.

Next, we evaluated the effect of RpoN on the activity of *PcrcZ* and *PcrcY* promoters. To this end, the transcriptional fusions *PcrcZ*-*gusA* and *PcrcY-gusA* were transferred to strain AErpoN, resulting in strains AErpoNZgus and AErpoNYgus. The activity of both promoters was suppressed in this genetic background, revealing their total dependence on RpoN. This result ruled out the functionality of the predicted σ^70^ promoters located in these regulatory regions under the tested conditions (Fig 5B).

Next, the levels of CrcZ and CrcY were determined by qRT-PCR in the RpoN-deficient strain in relation to the wild type strain. While the levels of CrcY showed a clear reduction, the abundance of CrcZ was 9-fold higher than in the presence of RpoN (Fig 5C).

Since transcription from *PcrcZ* was strictly dependent on RpoN and since the *PcrcZ-gusA* construction bore the overall intergenic *cbrB-crcZ* region, we investigated the existence of an alternative promoter, responsible for the detected *crcZ* transcripts in the RpoN-deficient strain.

### A *cbrB-crcZ* co-transcript and a processed form of CrcZ are present in *A*. *vinelandii*

**Fig 6:**
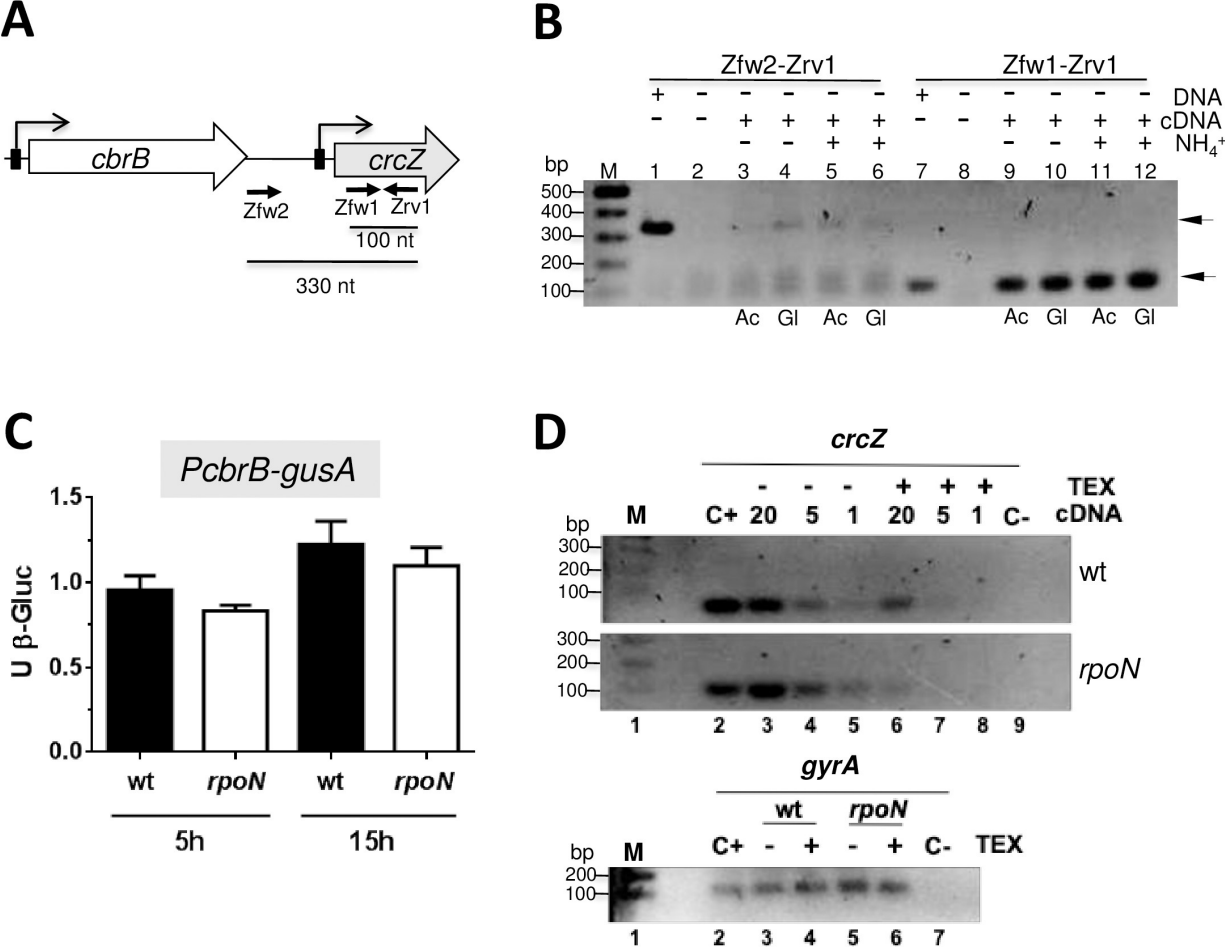
Identification of a *cbrB-crcZ* co-transcript in *A*. *vinelandii*. A. Representation of the *cbrB-crcZ locus* in *A*. *vinelandii*. The positions of the promoters is indicated, along with the oligonucleotides used for the reverse transcription-PCR (RT-PCR) assay of panel B. B. Identification of *crcZ* transcripts by RT-PCR. The wild-type strain AEIV was cultured in BAG medium with or without ammonium (NH_4_^+^), under acetate (Ac) or glucose (Gl) consumption. The total RNA was purified and used to generate cDNA using an oligonucleotide annealing within *crcZ* (Zrv1). The cDNA was PCR amplified with primer pairs Zfw2/Zrv1 or Zfw1/Zrv1, generating products of 330 and 100 bp, respectively. Control reactions using genomic DNA (lanes 1 and 7) or RT-PCR reactions in the absence of cDNA (lanes 2 and 8) are shown. M, DNA Molecular Weight Marker. C. The activity of the *cbrB* promoter (*PcbrB*) is RpoN-independent. Strain JG513 (*PcbrB*-*gusA*; wt) and its *rpoN*^*-*^ derivative AErpoNBgus, were cultured in BAG-N medium. Cells were harvested under acetate (5h) or glucose growing conditions (15 h), and the activity of ß-glucuronidase (ß-Gluc) was determined. D. Sensitivity of *crcZ* transcripts from the wild type strain AEIV (wt) or its isogenic *rpoN*^*-*^ mutant to the TEX enzyme. *crcZ* RT-PCR reactions were performed as in panel B, using primers pair Zfw1/Zrv1, and total RNA extracted from cells grown in BAG-N medium for 5h. When indicated, prior to the generation of the cDNA, RNA samples were treated with TEX. The amount of cDNA in nanograms used as a template for the PCR reaction in each experimental condition is indicated at the top. RT-PCR reactions using 20 nanograms of cDNA derived from the *gyrA* mRNA was used as an internal control, using primer pair gyrAfw/gyrArev (100 bp amplicon). Control reactions using genomic DNA (C+) or RT-PCR reactions in the absence of cDNA (C-) are shown. M, DNA molecular weight marker. The assay was repeated twice obtaining essentially the same results.

In order to test the possibility that some *crcZ* transcripts could actually come from readthrough transcription from the upstream *cbrB* gene, we designed a forward oligonucleotide annealing far upstream of the RpoN-dependent promoter of *crcZ* and a second one (reverse), located within *crcZ* (Fig 6A), which amplified a 330 bp amplicon. As a control, we also used a set of primers amplifying a 100 bp amplicon within *crcZ*. Reverse transcription PCR with total RNA from the wild type strain grown in BAG medium revealed the existence of a *cbrB-crcZ* co-transcript during both, acetate and glucose consumption in the presence or absence of ammonium (Fig 6B).

DNA sequence analysis of the *cbrB-crcZ locus* indicated the absence of potential promoters within the *cbrB* gene. In addition, the *cbrB-crcZ loci* from *A*. *vinelandii* and *P*. *putida*, including the *cbrB* regulatory region, are highly conserved (S1 Fig). Therefore, we investigated the presence of a constitutive *cbrB* promoter independent of RpoN. A transcriptional *PcbrB-gusA* fusion was constructed and introduced into the wild type strain AEIV and its *rpoN*^*-*^ derivative mutant AErpoN. The activity of *PcbrB* was monitored in diauxic acetate-glucose medium in the presence of ammonium. The levels of ß-glucuronidase activity from this *gusA* fusion were very similar regardless of the carbon source or the genetic background, thus showing a constant transcription pattern independent of RpoN (Fig 6C).

Next, the presence of a processed CrcZ sRNA in *A*. *vinelandii*, derived from the *cbrB*-*crcZ* co-transcript originated from the *cbrB* promoter, was investigated in the wild type strain and in the *rpoN*^*-*^ mutant. Total RNA was isolated from strains AEIV and AErpoN, cultured in BAG-N medium for 5 h; thereafter, it was treated with Terminator 5’-phosphate-dependent exonuclease enzyme (TEX, Epicentre). TEX specifically degrades RNAs having a 5’ monophosphate but not the primary transcripts with three phosphates at their 5’ end. Semi-quantitative RT-PCR was performed to assess the presence of *crcZ* transcripts resistant to the TEX treatment. As shown in Fig 6D, in RNA samples of the wild type strain, *crcZ* transcripts were detected and diminished 60% after TEX treatment. In agreement with our results presented in Fig 5C, in the RpoN-deficient strain the *crcZ* transcripts detected by this semi-quantitative method were more abundant than those of the wild type strain (Fig 6D). However, the amount of *crcZ* was reduced by 88% in this mutant after TEX treatment, confirming the prevalence of the processed variant of CrcZ (hereafter named CrcZ*) generated from the *cbrB-crcZ* co-transcription.

### CrcZ* is required for growth in the presence of acetate

In *P*. *putida* expression of CrcZ* *in trans* partially complemented the growth defect of the Δ*crcZ*Δ*crcY* double mutant in non-preferred carbon sources such as glucose or succinate [16]. We therefore sought to explore the role of CrcZ* in antagonizing the Hfq-Crc activity in *A*. *vinelandii*. In this bacterium, the genes *crcZ* and *crcY* seem to be essential as we were unable to isolate CrcZ- or CrcY-null mutants, despite the fact that the function of these sRNAs is presumably redundant [5]. This suggested that the unregulated activity of the protein complex Hfq-Crc is detrimental for cell growth. Thus, as an approach to explore the functionality of CrcZ*, the ability to use different carbon sources by the RpoN-deficient strain (mainly carrying the CrcZ* variant) was evaluated and was compared to that of strain CFB03, which was used as a negative control. Mutant CFB03 carries an insertional inactivation of *cbrB* with an Ω Sp resistance cassette (*cbrB*::Sp) [18]. The insertion of the Sp cassette occurred in the opposite orientation as that of *cbrB* transcription (S2 Fig) and therefore, would block *cbrB-crcZ* co-transcription. In addition, since the activity of *PcrcZ* and also that of *PcrcY* were RpoN dependent, this CbrB-null mutant would also lack primary *crcZ* and *crcY* transcripts. CFB03 was isolated on solid Burk’s medium with sucrose as the sole carbon source, as in *A*. *vinelandii* sucrose uptake and metabolism are not subjected to a strong repression by Hfq-Crc; although able to use this substrate, CFB03 shows a poor growth with respect to the wild type strain [18].

As mentioned before, the RpoN-deficient strain, AErpoN, was unable to use glucose, either in liquid Burk’s medium amended with glucose as the sole carbon source or in a combination with acetate (Fig 5A). This implies that CrcZ* is not able to relieve the repressing effect of Crc-Hfq under strong catabolite repressing conditions. Therefore, the functionality of CrcZ* (i.e. the ability to antagonize Crc-Hfq activity), in conditions of low-CCR (in the presence of organic acids) was evaluated.

For this purpose, the ability of strains AErpoN and CFB03 to grow on plates of solid Burk’s minimum medium amended with malate, fumarate or acetate as unique carbon sources was explored, looking for a condition where the CrcZ* variant present in the AErpoN strain, but not in the CFB03 mutant, would be sufficient for relieving the repressing activity of Crc-Hfq, allowing cell growth.

**Fig 7:**
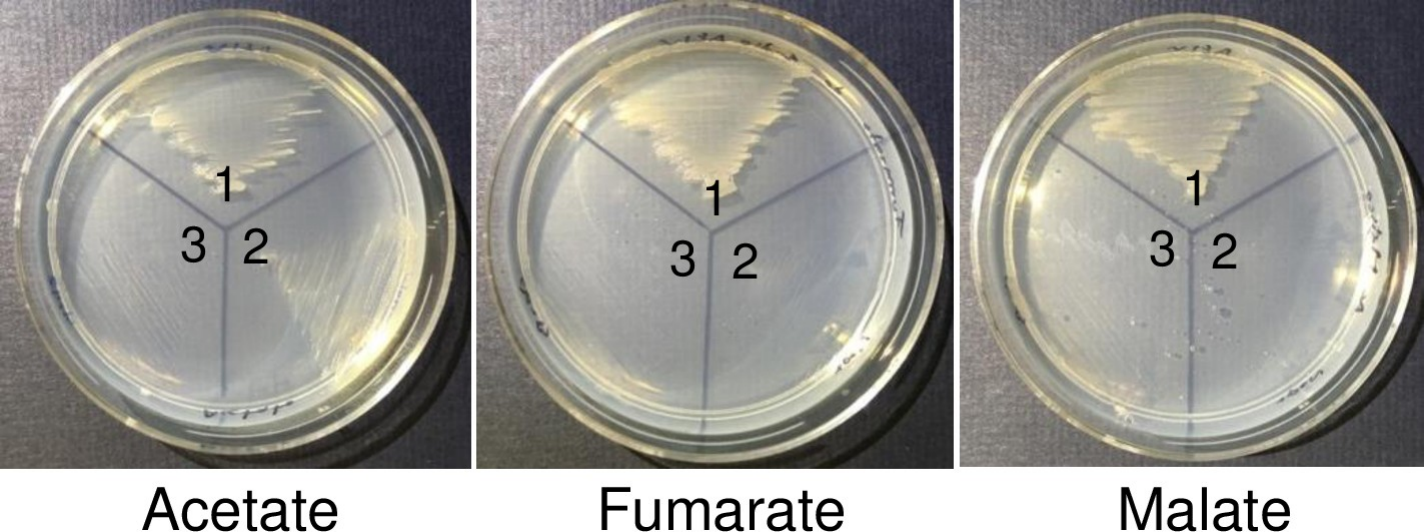
Growth of *A*. *vinelandii* strains on solid Burk’s medium. The wild type strain AEIV (1) and its isogenic RpoN- (2) and CbrB-null mutants (3) were cultured on plates of minimum Burk’s medium amended with 30 mM of acetate, fumarate or malate. The plates were incubated at 30°C /48 h.

As anticipated, the AErpoN mutant grew in the presence of acetate. However, it was unable to use either fumarate or malate, implying that under these conditions the levels of CrcZ* were not sufficient to counteract the activity of Hfq-Crc (Fig 7). In contrast, the CFB03 mutant, in which the levels of the primary *crcZ* and *crcY* transcripts are suppressed and those of the processed *crcZ* form are blocked, was unable to use these substrates. Altogether, these data indicated that the levels of CrcZ* in *A*. *vinelandii* have a functional relevance alleviating the repressing activity of the free Hfq-Crc on acetate utilization.

## Discussion

In this work, we studied the regulation of the expression of the sRNAs *crcZ* and *crcY* in the diauxic acetate-glucose growth. As in *Pseudomonas* spp., in *A*. *vinelandii* the activity of the *crcZ* and *crcY* promoters and the abundance of both sRNAs (Fig 1C) increased during glucose consumption, that is, when the less preferred substrate was being consumed. These higher levels of CrcZ and CrcY might be necessary to counteract the repressing effect of Hfq-Crc on *gluP* mRNA translation reported previously [5], since mutants unable to consume glucose (such as CbrA- or RpoN-deficient strains) showed basal expression levels of *crcZ* and *crcY*.

The scenario of CCR control was similar for non-diazotrophic conditions as in the presence of a fixed nitrogen source (ammonium), glucose consumption was prevented until acetate was totally exhausted. Similar results were reported previously for the *A*. *vinelandii* OP strain, which exhibited diauxie in a BAG-N medium [34]. The OP strain is a mutant unable to produce the exo-polysaccharide alginate as a result of an *algU*::IS mutation and for many years has served as a model for the study of *A*. *vinelandii*. *algU* encodes a homologue of the stress response sigma factor RpoE in *E*. *coli* [27]. We also showed that in the presence of ammonium, the relative levels of CrcZ and CrcY in the wild type strain AEIV increased once the acetate was exhausted. Taken together, these results indicated that, as in *Pseudomonas* spp., the levels of CrcZ and CrcY sRNAs modulate the strength of CCR in *A*. *vinelandii*. In line with this finding, expression of the *A*. *vinelandii crc* gene was similar in conditions of low (acetate) or strong (glucose) CCR (S3A Fig). In addition, low CCR conditions did not correlate with low expression levels of *hfq*, further corroborating that the levels of CrcZ and CrcY determined the strength of CCR in *A*. *vinelandii* (S3B Fig).

**Fig 8:**
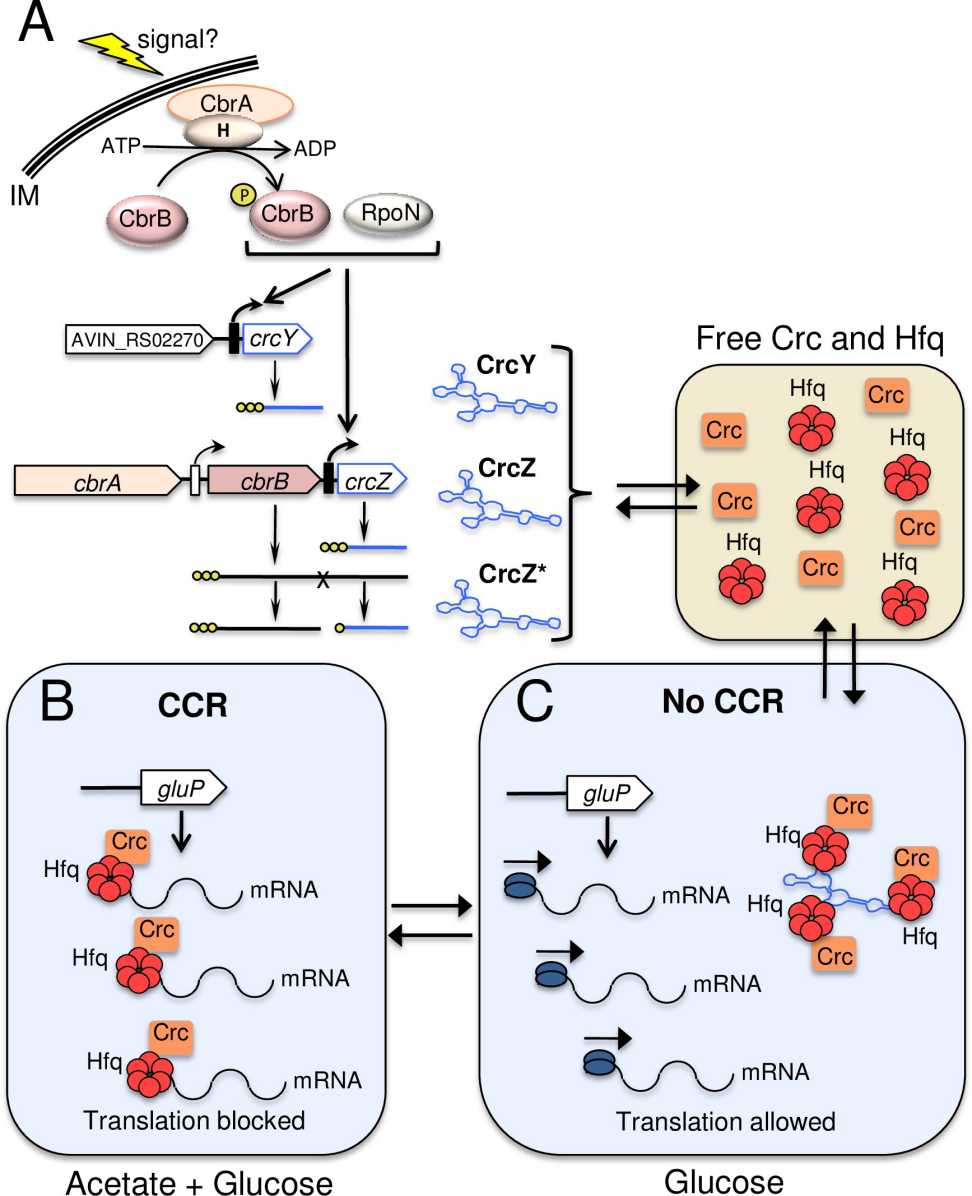
Model for the regulation of CCR in *A*. *vinelandii*, in diauxic glucose-acetate growth. A. The two-component system CbrA/CbrB is necessary for the expression of *crcZ* and *crcY* sRNAs genes from their RpoN-dependent promoters. A variant of CrcZ (CrcZ*) is produced from the processing of a *cbrB-crcZ* co-transcript, which is expressed from *PcbrB*. CrcZ, CrcZ* and CrcY sRNAs antagonize the translational repressing effect of Hfq-Crc on target genes. The phosphate groups at the 5’ end of *crcY*, *crcZ* and crcZ* transcripts are represented by yellow circles B. In the presence of both, acetate and glucose, translation of the *gluP* mRNA, encoding the glucose transporter, is inhibited by Hfq-Crc. C. This repression is alleviated as a consequence of the activation of the two-componente system CbrA/CbrB once the acetate is consumed, resulting in higher levels of CrcZ and CrcY, which sequester Hfq-Crc. For simplicity, other Hfq-Crc targets during glucose CCR are not shown [5]. Only three out of the six A-rich Hfq binding motifs of CrcZ/Y are represented to bind Hfq-Crc.

Our results also revealed that the promoters driving expression of *crcZ* and *crcY*, either in low or strong CCR, were RpoN-dependent as the activity of *PcrcZ* and *PcrcY* was abrogated in the RpoN-deficient strain (Fig 5B). This result is in agreement with the dependence of these promoters on the CbrA/CbrB two-component system, that upon phosphorylation, the response regulator CbrB acts as an enhancer binding protein of RpoN-dependent promoters. Despite the absence of a functional *PcrcZ* promoter, we detected *crcZ* transcripts in the RpoN-deficient strain in acetate growing cells (Fig 5C). Reverse transcription PCR analysis confirmed the existence of a CrcZ variant (named CrcZ*), resulting from the processing of the *cbrB-crcZ* cotranscript (Fig 6B). In support of this, our results from the primer extension assay revealed a second signal, which mapped at position -13, relative to the primary transcriptional start site (Fig 4B); this signal was constant under all the tested conditions and corresponded to a CrcZ sRNA similar in length to that of the processes CrcZ* detected in *P*. *putida* [16]. A proposed model for the regulation of CCR in *A*. *vinelandii* is presented in Fig 8.

In *A*. *vinelandii* CrcZ* was functional and necessary to sustain growth of the RpoN-deficient strain at the expense of acetate. However, this mutant did not grow using fumarate or malate, which are less preferred substrates with respect to acetate [3] (Fig 7). This result implies that the levels of CrcZ* in the RpoN-deficient strain were sufficient to antagonize the repressing activity of Hfq-Crc, allowing cell growth only at the expense of preferred carbon sources such as acetate.

Even though acetate was a preferred substrate for *A*. *vinelandii*, the inability of the CFB03 mutant to metabolize this carbon source implied that this substrate is subjected to a low CCR. In *A*. *vinelandii* the first two steps for acetate utilization comprise its phosphorylation by an acetate kinase (AckA) and its conversion to acetyl-CoA by the action of a phosphate acetyltransferase (Pta) enzyme. The generated acetyl-CoA is channeled through the glyoxylate shunt, converting this molecule to anapleurotic and gluconeogenic compounds [35]. The genome of *A*. *vinelandii* contains three *ackA* (*ack-1*, *ack-2* and *ack-3*), and only *ackA-1* showed a putative A-rich Hfq-binding motif at position -61 relative to the translational start codon (S4 Fig). In a previous report from our research group, we identified proteins induced during the *A*. *vinelandii* cell differentiation by comparing the proteome of vegetative vs. encysting cells [36]; while AckA-1 was only expressed during vegetative growth, AckA-3 was found exclusively in the differentiated cell. We were unable to identify AckA-2 in any of these conditions. On the other hand, *A*. *vinelandii* possesses two *pta* genes, *pta-1* and *pta-2*, located immediately downstream of *ackA-1* and *ackA-2*, respectively, however they did not show apparent Hfq-binding motifs. An analysis of the glyoxylate enzymes (malate synthase and isocitrate lyase), led us to identify a conserved A-rich Hfq-binding site at position +42 relative to the ATG translation initiation site of the isocitrate lyase-encoding gene AVIN_RS12975 (S4 Fig). Together, the results of this analysis further suggested that the utilization of acetate is subjected to CCR and identified the AckA-1 and the isocitrate lyase enzymes as potential targets of Hfq-Crc in *A*. *vinelandii*.

We previously reported that a CbrA-deficient strain was unable to use glucose despite its ability to grow using sucrose as a carbon source [5]. This result led us to propose that expression of the glucose transporter GluP, but not that of sucrose, was subjected to a strong CCR preventing the uptake of this substrate in CCR conditions. The RpoN-deficient strain phenocopied the growth of the *cbrA* mutant since it was originally isolated on plates of Burk´s-sucrose medium, but it was unable to grow at the expense of glucose in liquid Burk’s medium. These results highlight the importance of the primary CrcZ and CrcY sRNAs for glucose uptake. To our surprise, however, an incipient growth of the AErpoN and CFB03 mutants was detected on plates of solid Burk’s medium in the presence of glucose (S5 Fig). As GluP was also needed for the uptake of glucose on solid medium (S6 Fig), our data suggest that under this condition the *gluP* mRNA might escape from the translational repression of Hfq-Crc, and once inside the cell glucose is metabolized by the action of enzymes not subjected to CCR. In support of this idea, *A*. *vinelandii* has the uncommon characteristic of harboring large numbers of highly similar carbohydrate metabolism homologues (called synologues as they share >90% identity), which are proposed to confer adaptive benefits under certain environmental conditions [37]. In fact, only one of the two *eda* homologue genes encoding enzymes of the Entner-Doudoroff pathway was found to be a target of Hfq-Crc [5], whereas the two *edd* genes contained in the *A*. *vinelandii* genome lack potential A-rich Hfq binding sites. This flexible CCR control of glucose on solid growth medium might be associated with the germination of *A*. *vinelandii* cysts resistant to desiccation [38]. Under conditions of nutrient availability, particularly in the presence of glucose, the dormant cyst germinates originating two vegetative cells that resume growth. Therefore, such flexibility might represent adaptive benefits allowing successful germination and growth.

It is worthy to point out that subsequent subcultures of the CbrB- and RpoN-deficient strains on solid Burk’s-glucose medium rendered these strains able to utilize glucose in liquid medium, suggesting the generation of suppressor mutations; the identification and further characterization of such mutations remain to be investigated and would be important to identify new regulatory elements controlling the process of CCR in *A*. *vinelandii*.

A processed form of CrcY was reported for *P*. *putida* [13]. This variant is presumably derived from a large co-transcript originated from PP3539, encoding a homologue of the *P*. *aeruginosa* LiuR transcriptional regulator. In contrast to *P*. *putida*, convergent genes, encoding hypothetical proteins, flank *crcY* in *A*. *vinelandii*. Of note, we did not detect a processed form of CrcY in *A*. *vinelandii* as the levels of CrcY were suppressed in the RpoN- and in the CbrA-deficient strains.

In summary, our work represents the first study of the expression of the sRNAs *crcZ* and *crcY* in a nitrogen fixing bacterium and confirms their key role in determining the strength of CCR under diazotrophic and non-diazotrophic growing conditions. Furthermore, our results highlight the essential role of these sRNAs, either in their primary or processed form, in the vegetative growth of *A*. *vinelandii*, even for the utilization of preferred substrates such as acetate.

## Supporting information

S1 Fig Comparison of the *cbrB-crcZ loci* of *P*. *putida* and *A*. *vinelandii*. **(A)** Alignment of the *cbrB* regulatory region of *A*. *vinelandii* and *P*. *putida*. The location of the predicted σ^70^ promoters (-10 and -35 regions), based on the *P*. *aeruginosa cbrB* promoter reported previously [39], is indicated. The *cbrA* stop codon (TGA) as well as the *cbrB* ATG translational start codon are shown. **(B)** Alignment of the CrcZ regulatory region of *A*. *vinelandii* and *P*. *putida*. The location of the σ^54^ promoter (-12 and -24 regions) driving *crcZ* expression is indicated along with the transcription initiation site (+1) determined by primer extension in *A*. *vinelandii* (this work) and in *P*. *putida* [13].
(PDF)

S2 Fig The mutant CFB03 (*cbrB*::Sp) carries an Ω Sp resistance cassette inserted in the opposite orientation as that of *cbrB* transcription. **(A)** Genetic arrangement of the *cbrB locus* in the wild type strain AEIV and in mutant CFB03. Arrows indicate the direction of transcription. The location of the primers (represented by red arrows) used in panel B is shown. **(B)** PCR analysis to confirm the orientation of the Ω Sp insertion in mutant CFB03. Amplification of a 754 bp fragment corresponding to the wild type *cbrB* allele using DNA of the wild type strain AEIV (lane 1) or mutant CFB03 (lane 2) using primers *a* and *b*. A fragment of 550 bp corresponding to the 5’ region of *cbrB* in mutant CFB03 was amplified using primer *a* and primer *c* and as a template genomic DNA of this mutant (lane 3). As a negative control a PCR reaction using primers *c* and *b* was also included (lane 4), using CFB03 genomic DNA as a template. Construction of mutant CFB03 and the sequence of primers *a* (cbrB-F) and *b* (cbrB-R) was reported previously [5]. Primer *c*, (named SpFL-F (5n-GCCCTACACAAATTGGGAG-3C), anneals at the 3’ terminus of the Sp cassette. M, DNA ladder.
(PDF)

S3 Fig Levels of *crc* and *hfq* mRNAs under different CCR conditions. (**A**) Activity of the *crc* promoter using a *Pcrc-gusA* transcriptional fusion. The AEIV derivative carrying this construction, named AKJ01, was cultured in BAG medium. Cells were harvested after 5 (growth at the expense of acetate; gray bars) and 20 h (growth at the expense of glucose; black bars) in the absence (diazotrophy) or in the presence (non-diazotrophy) of ammonium. For the construction of strain AKJ01 (*Pcrc*–*gusA*), the regulatory region of *crc* (*Pcrc*) was PCR amplified using oligonucleotides crc-gus XB-F (5´-TCTAGAGATCACGTCGTCGACGATCAG-3´) and crc-gus SM-R (5´-CCCGGGAGGTCGGATCGTCCAGTTCG-3´). The resulting fragment (603 pb) spans the complete 5’ region of the *crc* gene and part of gene *pyrE*, located upstream of *crc*, and was sub-cloned into the pJET1.2/Blunt vector, rendering plasmid pJET::Pcrc. The *Pcrc* regulatory region was released with a double *XbaI-SmaI* digestion (sites recognized by these endonucleases were included in the designed oligonucleotides), and ligated to plasmid pUMATcgusAT, previously excised with the same enzymes. The resulting plasmid was named pUMAPcrc. The wild- type strain AEIV was transformed with pUMAPcrc previously linearized with the *NdeI* endonuclease, and Tc^r^ transformants were selected. An AEIV derivative carrying the *Pcrc*-*gusA* transcriptional fusion integrated into the chromosome was named AKJ01. The presence of the *Pcrc-gusA* construction was confirmed by PCR. (**B**) Quantification of *hfq* transcripts by qRT-PCR analysis. Total RNA was extracted from cells growing in diauxic BAG (diazotrophy) or BAG-N (non-diazotrophy) medium at the expense of acetate (5h; gray bars) or glucose (20 h; black bars). Oligonucleotides hfqqPCR-F (5’-CGTTCCGGTTTCCATCTATC-3’) and hfqqPCR-R (5’-CCATCTGGCTGACAGTGTTC-3’) were used. The bars of standard deviation from three independent experiments are shown.
(PDF)

S4 Fig Putative Hfq-Crc recognition sites in genes for acetate catabolism in *A*. *vinelandii*. Nucleotide sequence of the flanking region of the ATG translation initiation site of genes encoding the acetate kinase AckA-1 and the isocitrate lyase. The putative A-rich motifs recognized by the Hfq-Crc protein complex are shown. The structural region of the genes is indicated in red. The complete genome sequence of *A*. *vinelandii* DJ strain is available at https://www.ncbi.nlm.nih.gov/nuccore/NC_012560.1
(PDF)

S5 Fig Growth of *A*. *vinelandii* strains on solid Burk’s medium. The wild type strain AEIV (1) and its isogenic RpoN- (2) and CbrB-deficient strains (3) were cultured on plates of minimum Burk’s medium amended with 30 mM of sucrose or glucose as the sole carbon source. The plates were incubated at 30°C/48 h. The RpoN- and CbrB-deficient strains showed a poor cellular growth when compared to the wild type strain. However, the alginate-overproducing phenotype in the absence of either CbrB or RpoN rendered a mucoid colony of larger size that mask their growth defect. The positive effect of CbrB on alginate production was expected, based on a previous report [18]. In this context, the alginate-overproducing phenotype of the RpoN-deficient strain was also expected.
(PDF)

S6 Fig GluP is also needed for glucose uptake on solid medium. The GluP-deficient strain AHI30 (*gluP*::Sp) [5] was cultured on plates of Burk’s minimum medium amended with sucrose (BS) or glucose (BG) as the sole carbon source. The plates were incubated at 30°C for 48 h.
(PDF)
