# Supplementary material for: Expression of the sRNAs CrcZ and CrcY modulate the strength of carbon catabolite repression under diazotrophic or non-diazotrophic growing conditions in Azotobacter vinelandii
Source: PLoS One. 2018 Dec 13;13(12):e0208975. doi: 10.1371/journal.pone.0208975 (PMC6292655; doi:10.1371/journal.pone.0208975)
Supplement: S1 Fig — (A) Alignment of the cbrB regulatory region of A. vinelandii and P. putida. The location of the predicted σ70 promoters (-10 and -35 regions), based on the P. aeruginosa cbrB promoter reported previously [39], is indicated. The cbrA stop codon (TGA) as well as the cbrB ATG translational start codon are shown. (B) Alignment of the CrcZ regulatory region of A. vinelandii and P. putida. The location of the σ54 promoter (-12 and -24 regions) driving crcZ expression is indicated along with the transcription initiation site (+1) determined by primer extension in A. vinelandii (this work) and in P. putida [13]. (PDF) [file pone.0208975.s001.pdf]

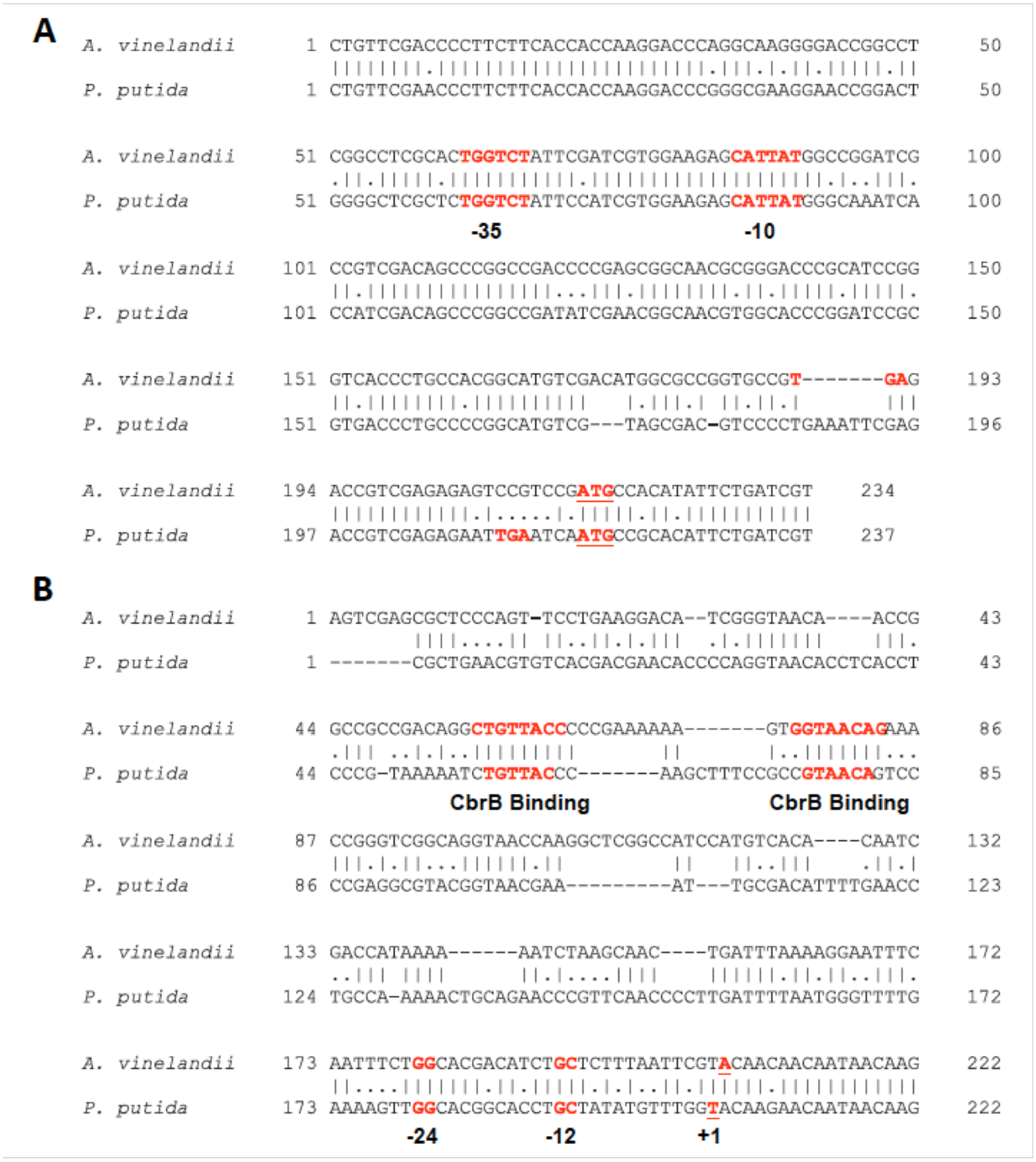

S1 Fig. Comparison of the *cbrB-crcZ loci* of *P. putida* and *A. vinelandii*. (A) Alignment of the *cbrB* regulatory region of *A. vinelandii* and *P. putida*. The location of the predicted σ^{70} promoters (-10 and -35 regions), based on the *P. aeruginosa cbrB* promoter reported previously [39], is indicated. The *cbrA* stop codon (TGA) as well as the *cbrB* ATG translational start codon are shown. **(B)** Alignment of the CrcZ regulatory region of *A. vinelandii* and *P. putida*. The location of the σ^{54} promoter (-12 and -24 regions) driving *crcZ* expression is indicated along with the transcription initiation site (+1) determined by primer extension in *A. vinelandii* (this work) and in *P. putida* [13].
