# Supplementary material for: Expression of the sRNAs CrcZ and CrcY modulate the strength of carbon catabolite repression under diazotrophic or non-diazotrophic growing conditions in Azotobacter vinelandii
Source: PLoS One. 2018 Dec 13;13(12):e0208975. doi: 10.1371/journal.pone.0208975 (PMC6292655; doi:10.1371/journal.pone.0208975)
Supplement: S2 Fig — (A) Genetic arrangement of the cbrB locus in the wild type strain AEIV and in mutant CFB03. Arrows indicate the direction of transcription. The location of the primers (represented by red arrows) used in panel B is shown. (B) PCR analysis to confirm the orientation of the Ω Sp insertion in mutant CFB03. Amplification of a 754 bp fragment corresponding to the wild type cbrB allele using DNA of the wild type strain AEIV (lane 1) or mutant CFB03 (lane 2) using primers a and b. A fragment of 550 bp corresponding to the 5’ region of cbrB in mutant CFB03 was amplified using primer a and primer c and as a template genomic DNA of this mutant (lane 3). As a negative control a PCR reaction using primers c and b was also included (lane 4), using CFB03 genomic DNA as a template. Construction of mutant CFB03 and the sequence of primers a (cbrB-F) and b (cbrB-R) was reported previously [5]. Primer c, (named SpFL-F (5n-GCCCTACACAAATTGGGAG-3C), anneals at the 3’ terminus of the Sp cassette. M, DNA ladder. (PDF) [file pone.0208975.s002.pdf]

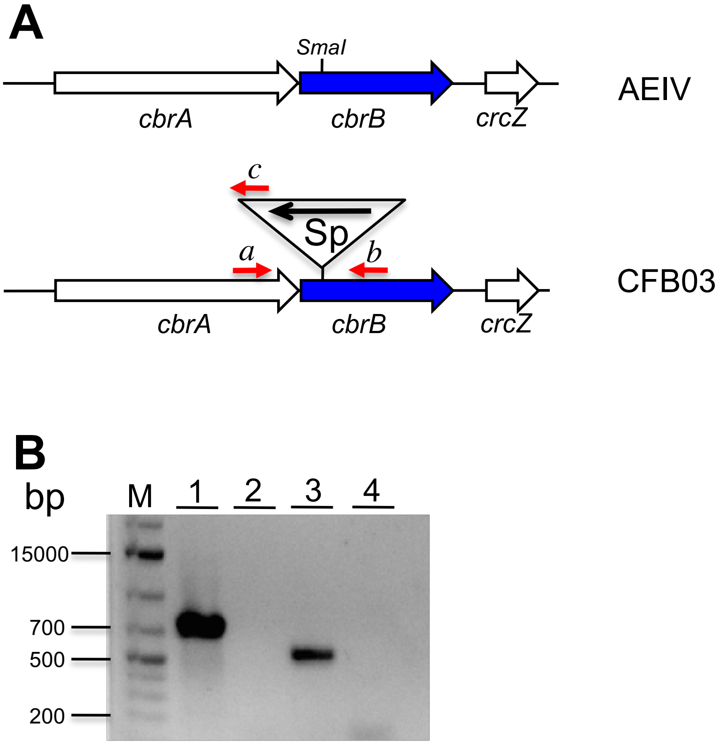

S2 Fig. The mutant CFB03 (*cbrB*::Sp) carries an Ω Sp resistance cassette inserted in the opposite orientation as that of *cbrB* transcription. (A) Genetic arrangement of the *cbrB locus* in the wild type strain AEIV and in mutant CFB03. Arrows indicate the direction of transcription. The location of the primers (represented by red arrows) used in panel B is shown. **(B)** PCR analysis to confirm the orientation of the Ω Sp insertion in mutant CFB03. Amplification of a 754 bp fragment corresponding to the wild type *cbrB* allele using DNA of the wild type strain AEIV (lane 1) or mutant CFB03 (lane 2) using primers *a* and *b*. A fragment of 550 bp corresponding to the 5' region of *cbrB* in mutant CFB03 was amplified using primer *a* and primer *c* and as a template genomic DNA of this mutant (lane 3). As a negative control a PCR reaction using primers *c* and *b* was also included (lane 4), using CFB03 genomic DNA as a template. Construction of mutant CFB03 and the sequence of primers *a* (cbrB-F) and *b* (cbrB-R) was reported previously [5]. Primer *c*, (named SpFL-F (5' GCCCTACACAAATTGGGAG - 3'), anneals at the 3' terminus of the Sp cassette. M, DNA ladder.
