# Supplementary material for: Expression of the sRNAs CrcZ and CrcY modulate the strength of carbon catabolite repression under diazotrophic or non-diazotrophic growing conditions in Azotobacter vinelandii
Source: PLoS One. 2018 Dec 13;13(12):e0208975. doi: 10.1371/journal.pone.0208975 (PMC6292655; doi:10.1371/journal.pone.0208975)
Supplement: S3 Fig — (A) Activity of the crc promoter using a Pcrc-gusA transcriptional fusion. The AEIV derivative carrying this construction, named AKJ01, was cultured in BAG medium. Cells were harvested after 5 (growth at the expense of acetate; gray bars) and 20 h (growth at the expense of glucose; black bars) in the absence (diazotrophy) or in the presence (non-diazotrophy) of ammonium. For the construction of strain AKJ01 (Pcrc–gusA), the regulatory region of crc (Pcrc) was PCR amplified using oligonucleotides crc-gus XB-F (5´-TCTAGAGATCACGTCGTCGACGATCAG-3´) and crc-gus SM-R (5´-CCCGGGAGGTCGGATCGTCCAGTTCG-3´). The resulting fragment (603 pb) spans the complete 5’ region of the crc gene and part of gene pyrE, located upstream of crc, and was sub-cloned into the pJET1.2/Blunt vector, rendering plasmid pJET::Pcrc. The Pcrc regulatory region was released with a double XbaI-SmaI digestion (sites recognized by these endonucleases were included in the designed oligonucleotides), and ligated to plasmid pUMATcgusAT, previously excised with the same enzymes. The resulting plasmid was named pUMAPcrc. The wild- type strain AEIV was transformed with pUMAPcrc previously linearized with the NdeI endonuclease, and Tcr transformants were selected. An AEIV derivative carrying the Pcrc-gusA transcriptional fusion integrated into the chromosome was named AKJ01. The presence of the Pcrc-gusA construction was confirmed by PCR. (B) Quantification of hfq transcripts by qRT-PCR analysis. Total RNA was extracted from cells growing in diauxic BAG (diazotrophy) or BAG-N (non-diazotrophy) medium at the expense of acetate (5h; gray bars) or glucose (20 h; black bars). Oligonucleotides hfqqPCR-F (5’-CGTTCCGGTTTCCATCTATC-3’) and hfqqPCR-R (5’-CCATCTGGCTGACAGTGTTC-3’) were used. The bars of standard deviation from three independent experiments are shown. (PDF) [file pone.0208975.s003.pdf]

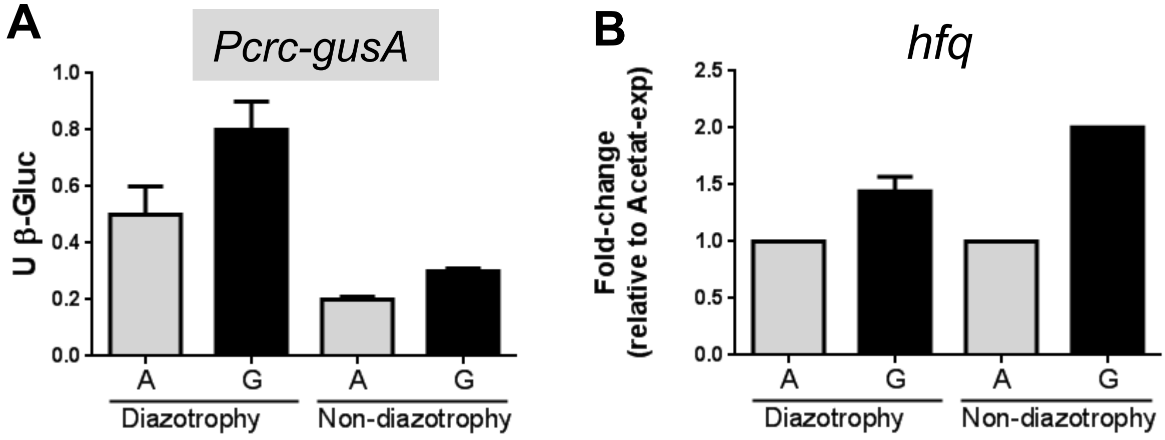


**S3 Fig. Levels of *crc* and *hfq* mRNAs under different CCR conditions.** (**A**) Activity of the *crc* promoter using a *Pcrc-gusA* transcriptional fusion. The AEIV derivative carrying this construction, named AKJ01, was cultured in BAG medium. Cells were harvested after 5 (growth at the expense of acetate; gray bars) and 20 h (growth at the expense of glucose; black bars) in the absence (diazotrophy) or in the presence (non-diazotrophy) of ammonium. For the construction of strain AKJ01 (*Pcrc–gusA*), the regulatory region of *crc* (*Pcrc*) was PCR amplified using oligonucleotides crc-gus XB-F (5´-TCTAGAGATCACGTCGTCGACGATCAG-3´) and crc-gus SM-R (5´-CCCGGGAGGTCGGATCGTCCAGTTCG-3´). The resulting fragment (603 pb) spans the complete 5' region of the *crc* gene and part of gene *pyrE,* located upstream of *crc*, and was sub-cloned into the pJET1.2/Blunt vector, rendering plasmid pJET::Pcrc. The *Pcrc* regulatory region was released with a double *XbaI-SmaI* digestion (sites recognized by these endonucleases were included in the designed oligonucleotides), and ligated to plasmid pUMATcgusAT, previously excised with the same enzymes. The resulting plasmid was named pUMAPcrc. The wild- type strain AEIV was transformed with pUMAPcrc previously linearized with the *NdeI* endonuclease, and $Tc^r$ transformants were selected. An AEIV derivative carrying the *Pcrc-gusA* transcriptional fusion integrated into the chromosome was named AKJ01. The presence of the *Pcrc-gusA* construction was confirmed by PCR. (**B**) Quantification of *hfq* transcripts by qRT-PCR analysis. Total RNA was extracted from cells growing in diauxic BAG (diazotrophy) or BAG-N (non-diazotrophy) medium at the expense of acetate (5h; gray bars) or glucose (20 h; black bars). Oligonucleotides hfqqPCR-F (5'-CGTTCCGGTTTCCATCTATC-3') and hfqqPCR-R (5'-CCATCTGGCTGACAGTGTTC-3') were used. The bars of standard deviation from three independent experiments are shown.
