# Supplementary material for: Expression of the sRNAs CrcZ and CrcY modulate the strength of carbon catabolite repression under diazotrophic or non-diazotrophic growing conditions in Azotobacter vinelandii
Source: PLoS One. 2018 Dec 13;13(12):e0208975. doi: 10.1371/journal.pone.0208975 (PMC6292655; doi:10.1371/journal.pone.0208975)
Supplement: S4 Fig — Nucleotide sequence of the flanking region of the ATG translation initiation site of genes encoding the acetate kinase AckA-1 and the isocitrate lyase. The putative A-rich motifs recognized by the Hfq-Crc protein complex are shown. The structural region of the genes is indicated in red. The complete genome sequence of A. vinelandii DJ strain is available at https://www.ncbi.nlm.nih.gov/nuccore/NC_012560.1 (PDF) [file pone.0208975.s004.pdf]

AckA-1 gene (AVIN_RS15720)

A-Rich motif

TGATTCTCGC CCATGTTCCA CAAGAACGCC GGGCAACCAT CCAATTACAA AAATTTGTGT

ackA-1

GGCCATCGAG CAATGATGCG CAGGGGCCAT ACACGACATG TTTCAGGAGT CCCAATGTCC

GCCCGCAATA TTCTGGTGAT CAACTGCGGT AGTTCATCAA TCAAGTTCGC CCTGGTCAAC

GAAGCGCAGA CGACCTTTCC CCTGCAGGGG CTGGCCGAGC GTCTCGGCAG TCCGGAAGCG

Isocitrate lyase gene (AVIN_RS12975)

TTCGACGTTT TTACCGCGTG TTGTCCGTAT CGATCATCGA CTACATGCAG GTTCAGGTGG

AVIN_RS12975

GAATCGCTCA ACCATTTCCA ACTTTGAAGG ATCGACCATG TCTGCATATC AAAACGAGAT

A-Rich motif

CAAGGCCCTT GCTGCCCTCA AAGAGAAGAA CGGCAGCGCC TGGAGCGCCA TCAACCCGGA

ATACGCCGCC CGCATGCGCA TCCAGAACCG TTTCAAGACC GGTCTGGACG TCGCCAAGTA

S4 Figure. Putative Hfq-Crc recognition sites in genes for acetate catabolism in *A. vinelandii*. Nucleotide sequence of the flanking region of the ATG translation initiation site of genes encoding the acetate kinase AckA-1 and the isocitrate lyase. The putative A-rich motifs recognized by the Hfq-Crc protein complex are shown. The structural region of the genes is indicated in red. The complete genome sequence of *A. vinelandii* DJ strain is available at https://www.ncbi.nlm.nih.gov/nuccore/NC_012560.1
