# Supplementary material for: Expression of the sRNAs CrcZ and CrcY modulate the strength of carbon catabolite repression under diazotrophic or non-diazotrophic growing conditions in Azotobacter vinelandii
Source: PLoS One. 2018 Dec 13;13(12):e0208975. doi: 10.1371/journal.pone.0208975 (PMC6292655; doi:10.1371/journal.pone.0208975)
Supplement: S5 Fig — The wild type strain AEIV (1) and its isogenic RpoN- (2) and CbrB-deficient strains (3) were cultured on plates of minimum Burk’s medium amended with 30 mM of sucrose or glucose as the sole carbon source. The plates were incubated at 30°C/48 h. The RpoN- and CbrB-deficient strains showed a poor cellular growth when compared to the wild type strain. However, the alginate-overproducing phenotype in the absence of either CbrB or RpoN rendered a mucoid colony of larger size that mask their growth defect. The positive effect of CbrB on alginate production was expected, based on a previous report [18]. In this context, the alginate-overproducing phenotype of the RpoN-deficient strain was also expected. (PDF) [file pone.0208975.s005.pdf]

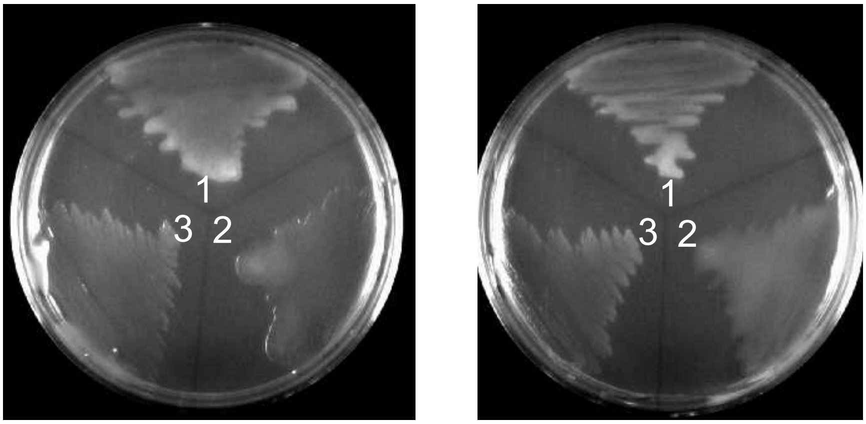

Sucrose Glucose

S5 Fig. Growth of *A. vinelandii* strains on solid Burk's medium. The wild type strain AEIV (1) and its isogenic RpoN- (2) and CbrB-deficient strains (3) were cultured on plates of minimum Burk's medium amended with 30 mM of sucrose or glucose as the sole carbon source. The plates were incubated at 30°C/48 h. The RpoN- and CbrB-deficient strains showed a poor cellular growth when compared to the wild type strain. However, the alginate-overproducing phenotype in the absence of either CbrB or RpoN rendered a mucoid colony of larger size that mask their growth defect. The positive effect of CbrB on alginate production was expected, based on a previous report [18]. In this context, the alginate-overproducing phenotype of the RpoN-deficient strain was also expected.
