# Supplementary material for: Expression of the sRNAs CrcZ and CrcY modulate the strength of carbon catabolite repression under diazotrophic or non-diazotrophic growing conditions in Azotobacter vinelandii
Source: PLoS One. 2018 Dec 13;13(12):e0208975. doi: 10.1371/journal.pone.0208975 (PMC6292655; doi:10.1371/journal.pone.0208975)
Supplement: S6 Fig — The GluP-deficient strain AHI30 (gluP::Sp) [5] was cultured on plates of Burk’s minimum medium amended with sucrose (BS) or glucose (BG) as the sole carbon source. The plates were incubated at 30°C for 48 h. (PDF) [file pone.0208975.s006.pdf]

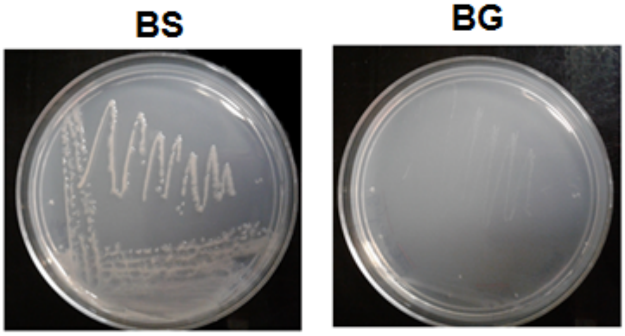

S6 Fig. GluP is also needed for glucose uptake on solid medium. The GluP-deficient strain AHI30 (*gluP*::Sp) [5] was cultured on plates of Burk's minimum medium amended with sucrose (BS) or glucose (BG) as the sole carbon source. The plates were incubated at 30°C for 48 h.
